# Burkitt lymphoma risk shows geographic and temporal associations with *Plasmodium falciparum* infections in Uganda, Tanzania, and Kenya

**DOI:** 10.1073/pnas.2211055120

**Published:** 2023-01-03

**Authors:** Kelly Broen, Joey Dickens, Rob Trangucci, Martin D. Ogwang, Constance N. Tenge, Nestory Masalu, Steven J. Reynolds, Esther Kawira, Patrick Kerchan, Pamela A. Were, Robert T. Kuremu, Walter N. Wekesa, Tobias Kinyera, Isaac Otim, Ismail D. Legason, Hadija Nabalende, Ian D. Buller, Leona W. Ayers, Kishor Bhatia, Robert J. Biggar, James J. Goedert, Mark L. Wilson, Sam M. Mbulaiteye, Jon Zelner

**Affiliations:** ^a^Department of Epidemiology, School of Public Health, University of Michigan, Ann Arbor, MI 48109; ^b^Center for Social Epidemiology and Population Health, School of Public Health, University of Michigan, Ann Arbor, MI 48109; ^c^Department of Statistics, University of Michigan, Ann Arbor, MI 48109; ^d^EMBLEM Study, St. Mary’s Hospital, Lacor, Gulu; ^e^African Field Epidemiology Network, Kampala, Uganda; ^f^EMBLEM Study, Moi University College of Health Sciences, Eldoret, Kenya; ^g^Academic Model Providing Access To Healthcare, Eldoret, Kenya; ^h^EMBLEM Study, Bugando Medical Center, Mwanza, Tanzania; ^i^Division of Intramural Research, National Institute of Allergy and Infectious Diseases, National Institutes of Health, Bethesda, MD 20892; ^j^Epidemiology of Burkitt Lymphoma in East-African Children and Minors (EMBLEM) Study, Shirati Health and Educational Foundation, Shirati, Tanzania; ^k^EMBLEM Study, Kuluva Hospital, Arua, Uganda; ^l^Division of Cancer Epidemiology and Genetics, National Cancer Institute, National Institutes of Health, Bethesda, MD 20892; ^m^Cancer Prevention Fellowship Program, Division of Cancer Prevention, National Cancer Institute, National Institutes of Health, Bethesda, MD 20892; ^n^Department of Pathology,The Ohio State University, Columbus, OH 43210

**Keywords:** endemic Burkitt’s lymphoma (eBL), Plasmodium falciparum malaria, non-Hodgkin lymphoma, East Africa, epidemiology

## Abstract

Endemic Burkitt lymphoma (eBL) is a pediatric cancer coendemic with malaria in sub-Saharan Africa, suggesting an etiological link between them. However, previous cross-sectional studies of limited geographic areas have not found a convincing association. We used spatially detailed data from the Epidemiology of Burkitt Lymphoma in East African Children and Minors (EMBLEM) study to assess this relationship. EMBLEM is a case–control study of eBL from 2010 through 2016 in six regions of Kenya, Uganda, and Tanzania. To measure the intensity of exposure to the malaria parasite, *Plasmodium falciparum*, among children in these regions, we used high-resolution spatial data from the Malaria Atlas Project to estimate the annual number of *P. falciparum* infections from 2000 through 2016 for each of 49 districts within the study region. Cumulative *P. falciparum* exposure, calculated as the sum of annual infections by birth cohort, varied widely, with a median of 47 estimated infections per child by age 10, ranging from 4 to 315 infections. eBL incidence increased 39% for each 100 additional lifetime *P. falciparum* infections (95% CI: 6.10 to 81.04%) with the risk peaking among children aged 5 to 11 and declining thereafter. Alternative models using estimated annual *P. falciparum* infections 0 to 10 y before eBL onset were inconclusive, suggesting that eBL risk is a function of cumulative rather than recent cross-sectional exposure. Our findings provide population-level evidence that eBL is a phenotype related to heavy lifetime exposure to *P. falciparum* malaria and support emphasizing the link between malaria and eBL.

## Significance

Endemic Burkitt’s lymphoma (eBL) is the most common pediatric cancer in malaria-endemic regions of sub-Saharan Africa and is universally fatal if untreated. Previous research has suggested an etiological link between malaria exposure and eBL. In this study, we used spatially detailed data on malaria parasite infection and eBL incidence in sub-Saharan Africa to assess the strength of this association. We found each additional 100 lifetime *P. falciparum* infections were associated with a 39% increase in age-specific risk of eBL, suggesting that malaria reduction may substantially reduce eBL incidence and mortality. Because the impact of malaria on eBL appears to be cumulative, evaluation of these efforts should account for lags between declines in malaria incidence and eBL incidence.

Endemic Burkitt lymphoma (eBL) is the most common pediatric cancer in equatorial regions of sub-Saharan Africa (SSA) where *Plasmodium (P.) falciparum* malaria is holoendemic (1). The cancer develops from mature germinal center B lymphocytes that have chromosomal translocations leading to the deregulated expression of the *MYC* gene (2). eBL has consistently been associated with Epstein Barr virus (EBV) and *P. falciparum* malaria (3), both of which are thought to explain the geographic concentration of eBL in malaria-endemic regions where EBV infection is ubiquitous (4, 5). The posited mechanism by which this occurs is an increase in chromosomal translocations, upregulated expression of *MYC*, and activation-induced cytidine deaminase activity, a mutator enzyme that promotes hypermutation in B cells (6, 7).

An association between country-level *P. falciparum* malaria and eBL incidence in SSA (8–10) was the first indicator of a potential etiological link (11). Other studies using spatiotemporal data to examine this relationship have yielded inconclusive results, in part due to the rarity of eBL cases, limited measurement of known confounders, and difficulty of reliably assessing *P. falciparum* infection incidence (8–10, 12–19).

We examined this association using *P. falciparum* and eBL incidence data from six regions of Uganda, Tanzania, and Kenya with generally elevated incidence of both. Data for 522 eBL cases were obtained from the Epidemiology of Burkitt Lymphoma in East African Children and Minors (EMBLEM) study, which enrolled eBL cases with associated geographical data in the study regions from 2010 to 2016 (20). Recent improvements in malaria surveillance, spurred by a renewed interest in malaria control and elimination (21, 22), have resulted in the production of high-quality, publicly available data on malaria incidence and infection pressure at a fine spatial scale, most notably from the Malaria Atlas Project (MAP) (23). We used the *P. falciparum* parasite rate (24) among children aged 2 to 10 y (*Pf*PR2-10) (a standard index of malaria transmission burden) data from 2001 to 2017 released by MAP at 5 sq. km. units resolution to estimate *P. falciparum* parasite prevalence in all birth cohorts by calendar year for each of the 49 districts in our dataset. Using the geographic birth cohort *Pf*PR2-10 data, we calculated the corresponding *P. falciparum* entomological inoculation rate (*Pf*EIR), which is the number of infectious mosquito bites (used interchangeably with inoculations) per person, per year, in all the geographical areas. *Pf*PR2-10 is directly proportional to *Pf*EIR in areas of high malaria transmission (25–28). Because the transmission efficiency of infectious mosquito inoculations, i.e., leading to blood-stage infection (with or without symptoms), is not 100%, we used a modeled estimate of a per bite transmission probability of 10% from the literature (27, 28) to estimate *P. falciparum* infection incidence given *Pf*EIR. This algorithm, shown in *SI Appendix*, Fig. S1, allowed us to estimate the expected number of cumulative *P. falciparum* infections for a child given their age, geographic location, and calendar-year risk and used this cohort and location *P. falciparum* infection metric to test the association of *P. falciparum* with eBL risk.

## Results

### Population at Risk, *P. falciparum* Infections, and eBL Cases.

The EMBLEM study areas and underlying population at risk are represented in *SI Appendix*, Fig. S2. We estimated the total person-time of children 0 to 15 y old (yo) observed from November 2010 to September 2016 was 59,793,102 person-years, including 29% from Kenya, 48% from Tanzania, and 23% from Uganda. *SI Appendix*, Fig. S3 shows the distribution of person-time, which included only Ugandan children under observation 2010 to 2011 and children from the three countries 2012 to 2016. The median age of children during the study period (2010 to 2016) was 8 yo in Kenya and Tanzania and 7 yo in Uganda, with a male:female ratio slightly greater than 1 in all countries.

The model estimated the number of annual *P. falciparum* infections per child by calendar year in the study areas 2000 to 2016 is shown in *SI Appendix*, Fig. S4, *Upper* panels. The graphs show that *P. falciparum* infections were higher in Uganda than in Kenya and Tanzania for all periods. *P. falciparum* infections had an overall decrease over calendar years of observation in all regions, albeit with some fluctuation in the trends. Infections in Uganda decreased from 41 infections per child in 2000 to 5 in 2013 and then increased to 24 in 2016, about half the burden in 2000. Infections in Kenya decreased from 21 per child per year in 2000 to 3 in 2005, where they remained at a plateau (~1.5) until 2013, followed by a slight upsurge to 7 in 2016. Infections in Tanzania declined from 8 to 1 between 2000 and 2016, without an upsurge. The long-term declines in annual *P. falciparum* infections per child coincide with the implementation of area-wide Government- and donor-supported malaria prevention programs in these countries (29, 30), and the upsurges noted in Uganda and Kenya coincide with the interruption of these programs, which led to a resurgence of infections noted in the data (29).

**Fig. 1.**
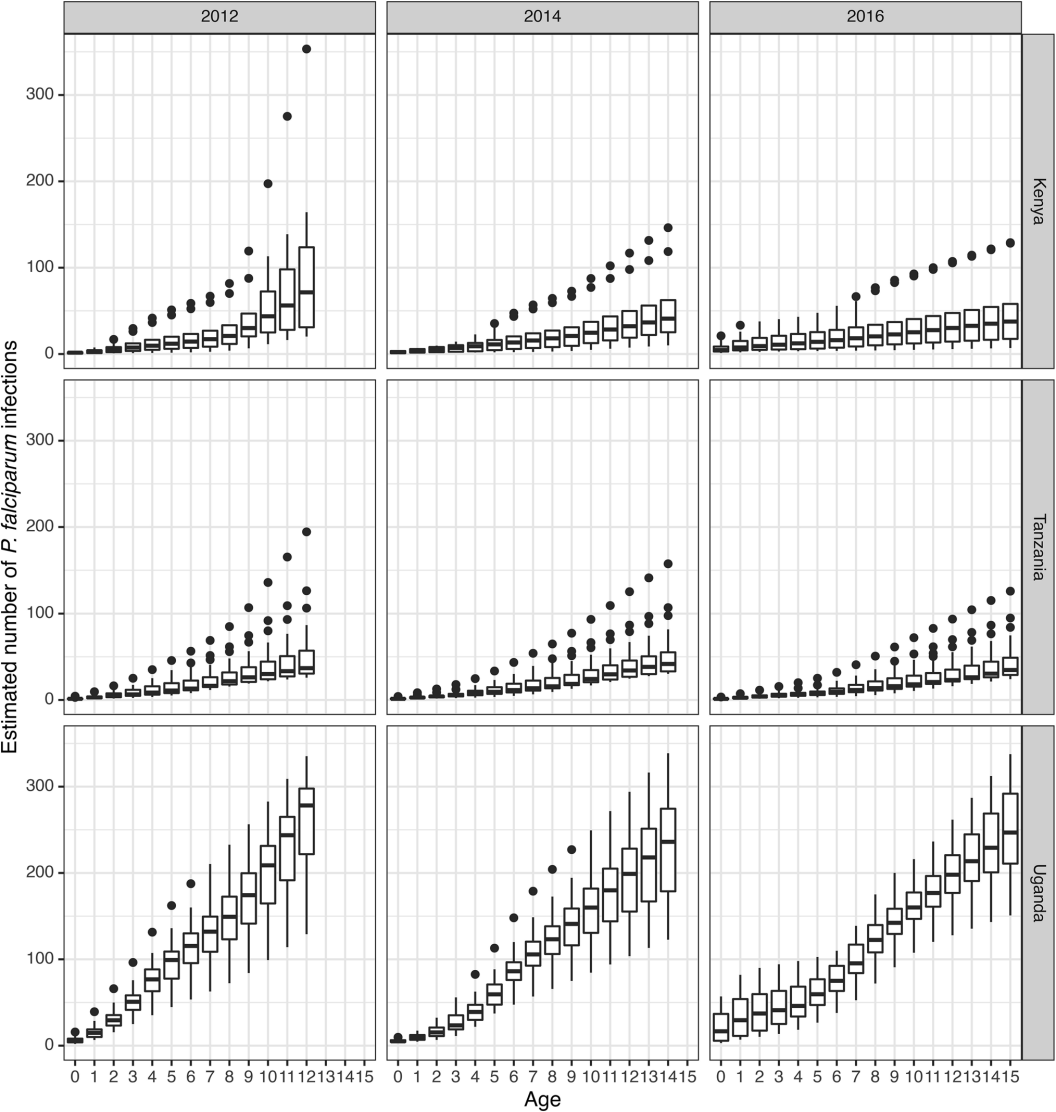
Cumulative estimated *P. falciparum* infections by age, country, and year. Box-and-whisker plots show the distribution of the cumulative number of expected *P. falciparum* malaria infections per year, for three example years, by age and country. Children born prior to 2000 were excluded from this analysis, which explains the absence of data for children 13 and over in 2012. The horizontal line of each box represents the median for each age, while the lower and upper bounds of the box represent the 25th and 75th percentiles, respectively. Vertical lines of each box represent the values from half of the 25th percentile to 1.5 times the 75th percentile, and dots outside this line show outlier values. The number of estimated infections varies both by geography and time. In 2012, the average 10 y-old had 61, 40, and 201 infections in Kenya, Tanzania, and Uganda, respectively. This changed to 33, 32, and 176 infections in 2014 and 36, 25, and 162 infections in 2016 in Kenya, Tanzania, and Uganda, respectively.

Fig. 1 shows the estimated cumulative number of *P. falciparum* infections for birth cohorts (0 to 15 y) during the three selected calendar years 2012, 2014, and 2016 for Uganda, Tanzania, and Kenya to illustrate the age-related increase in the number of infections seen typically in areas of holoendemic malaria (31, 32). Cumulative *P. falciparum* infection increases steeply with age in all countries and all calendar years.

However, the increase was steepest in Uganda, consistent with the higher annual rate of infections noted above (*SI Appendix*, Fig. S4*A*), and lower and comparable in Kenya and Tanzania. For example, the cumulative number of infections at ages 1, 5, and 12 y in 2012 was 16, 99, and 260 for Uganda; 3, 18, and 101 for Kenya and 3, 15, and 52 for Tanzania. Consistent with the declining incidence of annual infections described above (*SI Appendix*, Fig. S4, *Upper*), the cohort-based analysis shows that the cumulative *P. falciparum* infections in children older than 10 y were lower in each subsequent year for children of the same age (Fig. 1). For example, the cumulative infections by age 12 in Uganda decreased from 260 in 2012 to 196 in 2016, with the corresponding estimates for Kenya being 101 and 33 and for Tanzania being 52 and 42 for 2012 and 2016, respectively.

Of the 862 suspected eBL cases enrolled, 165 were excluded after pathology and clinical review, 46 were excluded because they were born prior to 2000 and would not have *Pf*PR2-10 MAP data, and 129 were excluded because they lacked complete geographic (*n* = 118) or demographic data (*n* = 11). Our analysis is based on a sample of 552 eBL cases with complete data required for inclusion in the spatial analysis (*SI Appendix*, Fig. S5), with 267 cases from Uganda, 102 from Tanzania, and 183 from Kenya. The mean age of included cases was 8.3 y (SD: ±3.7), with 63% of cases being male. There were no age and sex differences between the excluded and included cases.

**Fig. 2.**
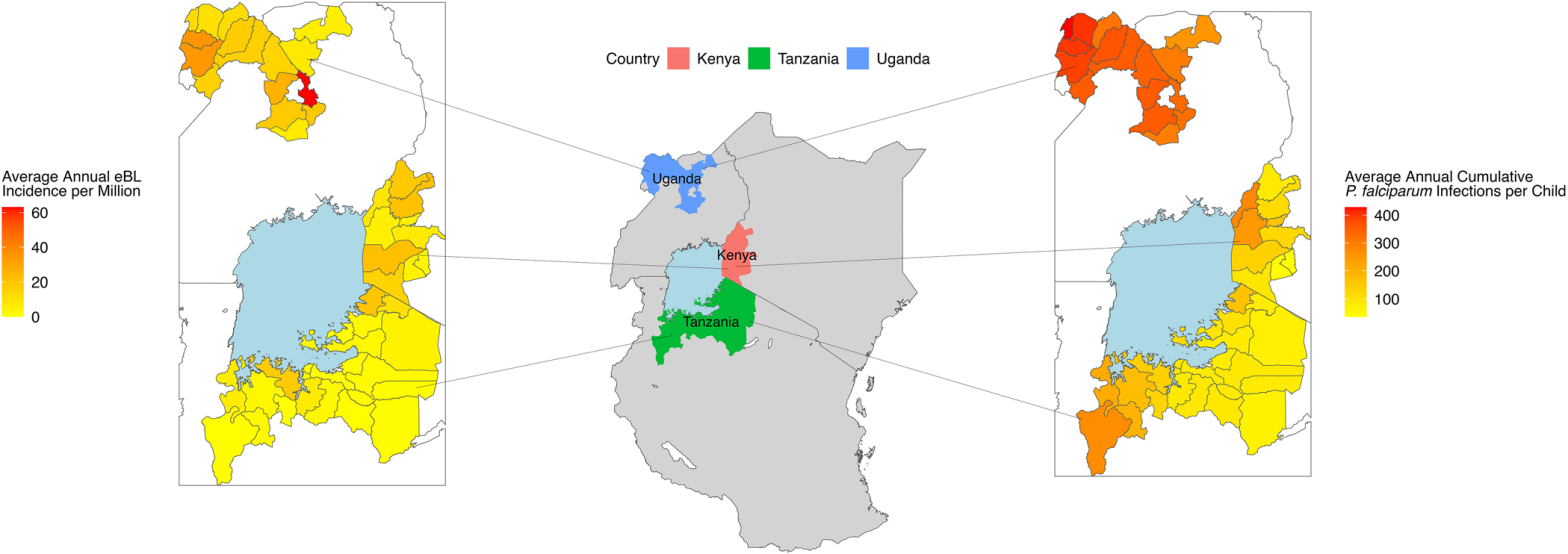
Spatial distribution of cumulative annual *P. falciparum* infection and annual eBL incidence. On the left is the average annual estimated number of cumulative *P.* falciparum infections from 2000 to 2016 by country and district for the areas included in the EMBLEM study. Darker colors indicate higher estimated number of cumulative infections, indicating greater average *P.* falciparum infection pressure. On the right is the total eBL incidence by country and district for the areas included in the EMBLEM study. Data are from 2010 through September 2016 in Uganda and 2012 through September 2016 in Kenya and Tanzania. Darker colors indicate higher total eBL incidence. In the center are the study regions colored by country, and Lake Victoria is colored blue. Average annual cumulative *P.* falciparum infections and the average eBL incidence are strongly correlated, with a Spearman correlation coefficient of 0.58 (95% CI: 0.34 to 0.70).

Overall, the average eBL annual incidence for the three countries combined was 9.4 cases per million person-years (Table 1). eBL incidence was 1.7 times higher in males than females (95% CI: 1.5 to 2.1) and incidence in Uganda was 1.8 times that of Kenya (95% CI: 1.5 to 2.3) and 5.3 times that of Tanzania (95% CI: 3.9 to 7.3). There was district-level variation of incidence within the countries (Fig. 2). *SI Appendix*, Fig. S4 (*Lower*) shows the eBL incidence in the three countries, suggesting a slight increase in eBL incidence during the first 1 to 2 y at the beginning of the EMBLEM study in each country, followed by a downtrend in all countries (Table 1). Overall, eBL incidence declined by about 9.6% (95% CI: 3.2 to 16.5%), but the estimate is imprecise.

**Table 1.** Descriptive statistics of eBL incidence per million in Kenya, Tanzania, and Uganda from 2010 to 2016

		Kenya	Tanzania	Uganda	Total	
N		183	102	267	552	
Incidence per million person-years		10.63	3.51	19.82	9.23	*P* < 0.01
Sex						
	Male	14.74 (127)	4.18 (61)	23.85 (163)	11.69 (351)	
	Female	6.51 (56)	2.82 (41)	15.66 (104)	6.76 (201)	*P* < 0.01
Age						
	0–4	4.46 (24)	1.56 (14)	5.87 (23)	3.34 (61)	
	5–9	12.77 (93)	4.85 (57)	24.29 (146)	11.82 (296)	
	10–15	14.48 (66)	3.71 (31)	27.65 (98)	11.84 (195)	*P* < 0.01
Year						
	2010			85.84 (4)	85.84 (4)	
	2011			19.85 (37)	19.85 (37)	
	2012	11.62 (16)	5.28 (14)	16.90 (35)	10.66 (65)	
	2013	15.32 (61)	3.45 (24)	28.45 (66)	11.39 (151)	
	2014	12.65 (55)	4.22 (32)	15.85 (41)	8.82 (128)	
	2015	7.57 (36)	2.92 (24)	17.65 (51)	6.99 (111)	
	2016	5.44 (15)	2.17 (8)	19.45 (33)	6.87 (56)	*P* < 0.01
Median Annual No. Malaria Infections (standard deviation)		2.12 (4.13)	1.35 (0.75)	7.34 (11.20)	2.72 (8.35)	*P* < 0.01

Incidence is calculated per million with the number of cases in parenthesis. *P* values were computed using one- and two-way ANOVA.

### Spatial Clustering of eBL and Cumulative *P. falciparum* Infection.

Fig. 2 shows the spatial patterns of the average annual *P. falciparum* infections and eBL incidence for 49 districts in the study area. The annual *P. falciparum* infections and the average eBL incidence are strongly correlated, with a Spearman correlation coefficient of 0.58 (95% CI: 0.34 to 0.7). Univariate Moran’s local indicators of spatial autocorrelation (LISA) analyses suggest spatial clustering of both total eBL incidence and cumulative estimated *P. falciparum* incidence over the study period. The significant univariate LISA values for both eBL incidence and cumulative estimated *P. falciparum* incidence, calculated by calendar year, were then summed over the observation period (2010 to 2016) and demonstrated strong clustering patterns of both metrics; however, the spatial overlap between clusters of cumulative *P. falciparum* incidence and eBL incidence was imperfect (*SI Appendix*, Fig. S6).

Bivariate Moran’s *I* LISA values for average cumulative *P. falciparum* and average eBL incidences showed 4 clusters with significant concordant (high–high or low–low) values and 3 with significant discordant values (high–low) (*SI Appendix*, Fig. S7). However, bivariate Moran’s *I* LISA calculated by year for annual cumulative *P. falciparum* incidence and eBL incidence only showed discordant pairs, with 7 discordant bivariate Moran’s *I* LISA values and 0 concordant values.

### Association between Lifetime Malaria Exposure and eBL.

**Fig. 3.**
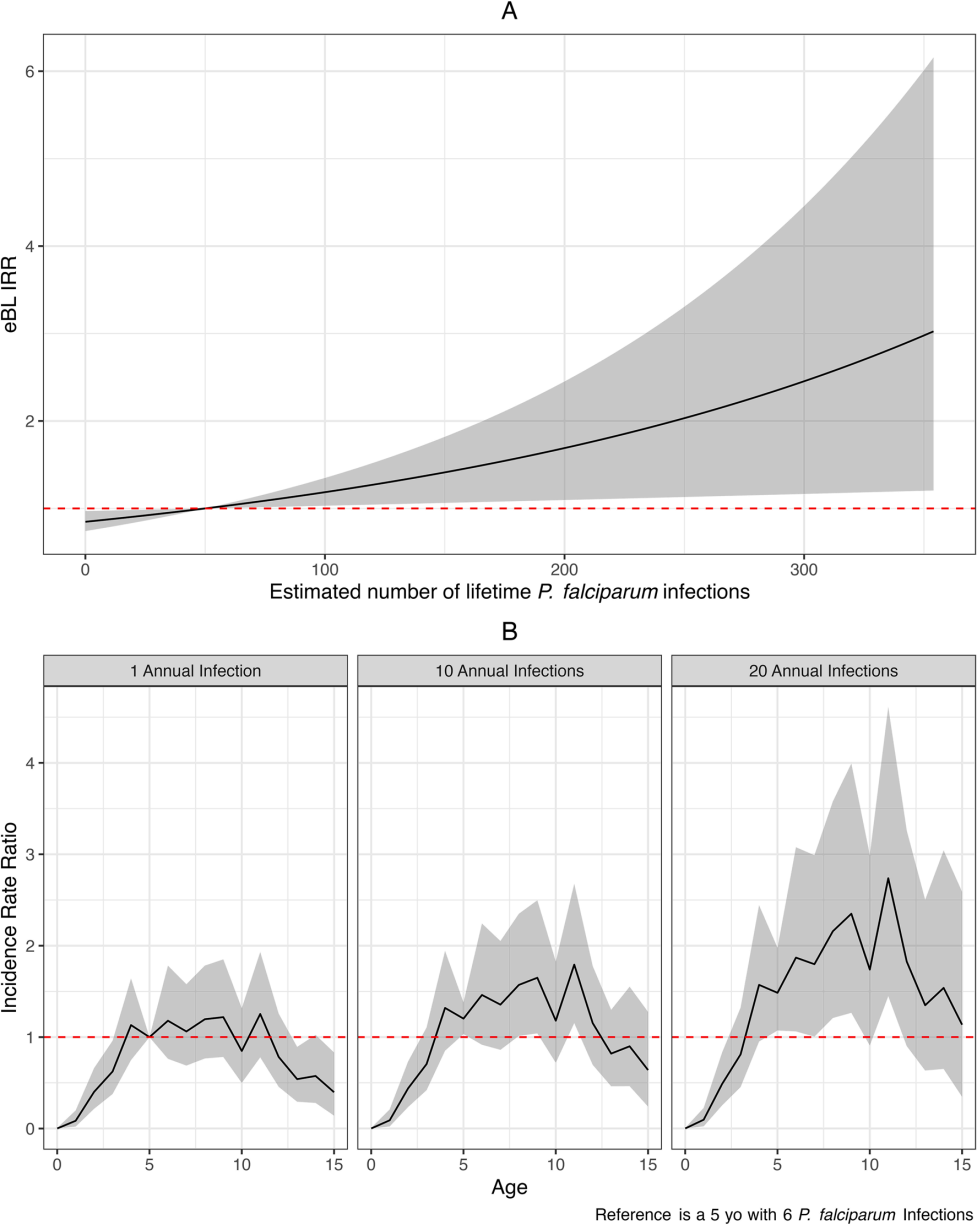
Risk of eBL by age and cumulative *P. falciparum* incidence. Panel *A* shows the IRR of eBL at all observed levels of estimated lifetime cumulative *P. falciparum* infections compared to 50 estimated lifetime *P. falciparum* infections (where the red dotted line crosses the slope), which is approximately the average number of estimated infections of the study population. The risk of eBL increases 39% for every 100 *P. falciparum* infections, with 150 estimated lifetime infections corresponding to an IRR of 1.39 when compared to an individual with 50 estimated lifetime malaria infections, holding all other characteristics the same. yo, year-old. In panel *B*, each plot shows the IRR of eBL for different age groups as the estimated number of lifetime *P. falciparum* infections increases. In each panel of the figure, solid lines indicate the IRR for eBL associated with both age and increasing exposure to *P. falciparum* infection. In panel 1, for every 1-y increase in age, cumulative *P. falciparum* exposure increases by 1 infection. Panels 2 and 3 demonstrate the increase in risk when infections increase to 10 and 20 per each year of life. IRRs in each panel are presented relative to 6 cumulative infections at age 5, or how many infections a child would have at the midpoint of age 5, assuming one infection per year, to allow comparison of both age- and exposure-specific differences in eBL risk.

We used a negative binomial regression model to explore the relationship between estimated lifetime *P. falciparum* incidence and annual risk of eBL. The model converged, with the $\hat{R}$ of all coefficients less than 1.1. The incidence rate ratio (IRR) of eBL increased by 39% (95% CI: 6.1 to 81.0%) for every 100 additional cumulative *P. falciparum* infections, as illustrated in Fig. 3*A*. In *SI Appendix*, Fig. S8, we present a sample of age-specific risks to illustrate how these risks vary with age. The probability of successful *P. falciparum* infection varies with age as a function of exposure to infectious mosquitoes and acquired immunity to *P. falciparum*, which increases with age. Thus, comparing the IRR to that of a 10 yo with 100 cumulative *P. falciparum* infections, we see that the IRR for eBL was not significantly above unity before age 5 and after age 11, regardless of cumulative *P. falciparum* burden but was significantly elevated between age 5 and 11 y. In Fig. 3*B*, we repeated the IRR analysis assuming a range of plausible fixed annual in *P. falciparum* infection(s) for each year of life (assumed to occur at the midpoint of the year, for birth cohorts 0 to 15 y). Compared to a child who accumulated 6 infections by age 5 (the lightest infection plausible in our study area), we found that eBL risk rises significantly above unity for ages 5 to 11 y for 10 and 20 annual infections, and that the risk in that fixed age group is higher for 20 infections than 10 infections. Alternative models, using annual estimated number of *P. falciparum* infections with different lag periods (0 to 10 y) before eBL onset, which are more similar to previous research (5, 9, 33) were less informative. Models considering prior *P. falciparum* incidences for different lag periods individually showed marginally decreased eBL risk for models with 0- and 1-y lag periods included, while those with 9- and 10-y lag periods were associated with elevated eBL risk (*SI Appendix*, Fig. S9). These findings may demonstrate that a single year’s measure of *P. falciparum* is inadequate to represent the lifetime *P. falciparum* burden, leading to nondifferential misclassification of the exposure and biasing results toward the null. This suggests that an individual’s cumulative or lifetime *P. falciparum* exposure, rather than a cross-sectional metric of annual infection burden, is the more relevant predictor of eBL incidence. Finally, compared to models with any or all lag periods, the model employing a cumulative metric of *P. falciparum* exposure explained more of the spatial variation in eBL incidence, as evidenced by smaller magnitude and lower variation of EA-level random intercepts.

## Discussion

Our results indicate a strong positive relationship between the cumulative number of *P. falciparum* infections and risk of developing eBL in children in Uganda, Tanzania, and Kenya. Also, the eBL risk was higher with a higher infection burden in children of a fixed age group (Fig. 3*B*). Importantly, the risk peaked and was significant between 5 and 11 y of age, providing insights into epidemiological patterns of eBL that have hitherto been difficult to explain. First, eBL is rare in children < 3 y of age, although this is the age when children are most susceptible to malaria. Our results suggest that unlike malaria, whose symptomatology is related to uncontrolled proliferation of parasites resulting in high-density infections in young children who are nonimmune, eBL appears to be related to repeated exposure to parasites resulting from hundreds of unique infection episodes, which are unlikely to be attained by children before age 3 y (34, 35).

Second, it is known that the age mode for eBL is 6 to 8 y. Our finding that malaria-related risk for eBL peaks between 5 and 11 y and decreases thereafter (an inverted U-shaped relationship between age and eBL risk) provides a rational explanation to link cumulative exposure of *P. falciparum* infections to the age mode of eBL. We hypothesize that naturally acquired immunity to malaria is both a risk factor (i.e., increases the overall survival from malaria whereby more patients would be alive and at risk of eBL) and a protective factor in older children, perhaps by decreasing the risk of successful infection given inoculation (36, 37). However, other developmental or environmental mechanisms may also explain this inverted U-shaped relationship.

Our analysis has several strengths that distinguish it from earlier investigations of the link between malaria and eBL. First, we used fine-scale, high-quality, large-area malariometric data available through the MAP (23) and EMBLEM’s granular eBL data from the same regions (20) to perform a larger, in-depth comprehensive longitudinal assessment of the relationship between *P. falciparum* exposure and eBL than has been possible to-date. We derived multiple metrics of *P. falciparum* infection (annual, lifetime, lagged infections) using computational methods, the established relationships between MAP estimates of *Pf*PR2-10 and *Pf*EIR, and assumptions about transmission efficiency (27, 28). Previous studies have been small, measured eBL on a coarse temporal and spatial scale (8, 9), modeled eBL incidence without explicitly modeling *P. falciparum* incidence (8, 18, 38), or used nonconcurrent large-area geographical data of malaria intensity (9). Although other studies have investigated the etiological link between *P. falciparum* and eBL using antibody markers of *P. falciparum* infections [such as IgG antibodies (4, 33)], those studies provide no insights into lifetime versus cross-sectional malaria exposure, spatial, temporal, and age-specific patterns of the cumulative number of *P. falciparum* infections. These metrics derived through our computational and integrative approach allowed for greater insight into the historical burden of infection.

Using these derived metrics of *P. falciparum* infections, parameterized as either the annual infections per child per year; or the sum of infections per child of a given age, location, and calendar year; or lagged cross-sectional infections, we found a strong relationship between eBL and the cumulative number of infections, which peaked between 5 and 11 y of age. Overall, children who attain roughly 100 or more infections experience an elevated risk for eBL; however, few children below 5 y attain this considerable number of infections. Conversely, eBL risk decreases below significance despite the continuing accumulation of infections. Since repeated *P. falciparum* infection elicits naturally acquired immunity, we hypothesize that eBL risk initially increases as the number of infections increases, perhaps in proportion with genetically complex *P. falciparum* infections. However, these infections elicit naturally acquired immunity (36), which ultimately depresses successful infections and eBL risk in older children.

The ubiquity of malaria in endemic regions, the relative rarity of eBL, and the longtime it takes to develop eBL make it challenging to establish an etiological relationship between *P. falciparum* exposure and eBL using cohort studies. Thus, prior studies have relied on cross-sectional designs to show associations between serological indicators of *P. falciparum* infection, such as IgG antibody titers (39); however, such results may not reflect antibody changes that occur before but those that occur after disease onset (40). While our bivariate Moran’s *I* analysis noted clustering of eBL and cumulative *P. falciparum* incidence, it did not consider confounding factors such as underlying age and sex distributions and may therefore miss important associations.

Nonetheless, this analysis demonstrates the importance of time scale when assessing the relationship between cumulative *P. falciparum* infections and eBL incidence. Our analyses that used only annual estimates of *P. falciparum* incidence further show the importance of total lifetime burden rather than recent time scale exposures, by replicating the ambiguous results of previous studies based on similar measures. While *P. falciparum* incidence from 9 and 10 years prior was associated with increased eBL risk, these results are difficult to interpret causally because eBL incidence peaks in ages too young to have exposures from 9 and 10 y ago. Our results add to previous findings (4) by clearly illustrating how cumulative, lifetime *P. falciparum* exposure can explain spatial variation in the geographic, temporal, and age-specific patterns of eBL incidence in SSA.

Our investigation also provides mechanistic insights into the epidemiology of eBL and malaria as a putative risk factor (8, 9). The model using only the prior year’s *P. falciparum* burden attributed much of the variation in eBL to district- and country-level effects, which provides no mechanistic insight. Using the cumulative metric of *P. falciparum*, the district- and country-level effects become much smaller, and the cumulative infections variable has a stronger relationship with variation in eBL incidence. Finally, our analysis suggests that the apparent “dose–response” relationship between *P. falciparum* exposure and eBL risk suggested by our analysis may also shed light on within-host processes leading to the development of eBL. Specifically, the high cumulative number of *P. falciparum* infections implies that robust naturally acquired immunity is developed before eBL and contributes to overall survival from malaria.

However, survival is gained at a cost of increased exposure to low-grade infections, which likely trigger abnormalities that initiate eBL onset (39). The chromosomal translocations involving *MYC*, believed to be early or initiating events in eBL pathogenesis, have been documented to increase with *Plasmodium chabaudi* infection in mouse studies (6) and may also occur in humans exposed to high cumulative number of *P. falciparum* infections.

Our results rest on several assumptions. First, we assume that most BL cases in the study area presented to a hospital at some point during their illness and that when they did, they were referred to the study hospitals as stressed in study BL health messages (41). We acknowledge that some eBL cases could have been treated in nonmedical settings, could have presented late and declined referral, or died before referral or diagnosis, which would result in incomplete ascertainment of cases. The EMBLEM study implemented community-wide public-awareness campaigns to reduce missing cases. Our finding that eBL case incidence increased during the 1 to 2 y after introducing the study suggests that health communication about eBL may have been effective, and to the extent it was successful, it likely reduced biased ascertainment that was present before area-wide awareness about eBL was introduced in the study area (42). While it is theoretically possible that we missed cases that were directly referred to tertiary-level care centers, which are located far away in the capital cities, previous reports indicated that most cases in the remote areas where the study was conducted were treated at local hospitals rather than at tertiary hospitals (4, 42). While it is impossible to describe cases not captured by these efforts, they likely lived further from hospitals where less resources also lead to greater malaria burden, which should contribute to an under-estimate of the strength of association between *P. falciparum* infection pressure and eBL incidence (39, 43).

We also note potential limitations of using *Pf*PR2-10 data from MAP to estimate malaria burden in children 0 to 15 yo. Although modeled from multiple sources of malariometric data accounting for each methodology’s sensitivity (23), our assumptions that all children in a given district are exposed to a stable malaria exposure risk for the entire year and that children reside in the same area for the year are necessary simplifications. These assumptions are reasonable because census data showed little change in overall district-level populations by age and sex during the 7-y study period; unobserved migration patterns between districts with different malaria infection pressures could bias our results. Another simplifying assumption is that each inoculation is in association with a transmission probability of 10%, which also assumes an average mosquito sporozoite load greater than 1,000. These assumptions are reasonable based on available field data (27, 28). Future studies utilizing residential history data incorporating mobility as well as actual sampling of mosquitoes in the field are promising areas of research to refine dimensions of variation in *P. falciparum* exposure discussed above.

Because it is not possible to measure the true number of asymptomatic *P. falciparum* infections a child will have before eBL, our analysis used the correlation between *Pf*EIR and *Pf*PR2-10 to estimate the sporozoite inoculation rate of children in the study population (25) and assumed a transmission efficiency of 10%, i.e., 10% of the inoculations lead to blood-stage infection (44, 45). The direct proportionality to the cumulative *P. falciparum* incidence means that assuming a different rate would not change the direction or shape of the relationship between cumulative *P. falciparum* incidence and eBL. We note that our analysis is unable to account the for interaction between *P. falciparum* infection with EBV, which is another risk factor for eBL (13). However, EBV infection is ubiquitous and is established during the first year of life among children in our study area (46).

To conclude, using high-resolution long-term serial malaria exposure data and granular eBL case data from well-defined geographic regions in Kenya, Tanzania, and Uganda, we showed geographic, temporal, and age-related associations between lifetime exposure to *P. falciparum* and eBL risk. Various analyses provided evidence that a greater *P. falciparum* burden increases eBL risk and justifies efforts to emphasize the link between malaria and eBL in public health messages. In view of the expanding interest in malaria control and elimination, these findings suggest that aspirations to reduce eBL mortality and eliminating life to eBL could be enhanced by linking eBL to antimalaria interventions. Because eBL occurs in countries currently designated as high-burden [for example Uganda ranks among the top 5 countries ranked by malaria cases and deaths (47)], high-impact areas for malaria intervention, additional reduction in risk for eBL could be emphasized as part of the cost-benefit analysis of malaria prevention and treatment campaigns in countries with holoendemic malaria and high eBL burden.

## Materials and Methods

### Study Population.

The study population comprised children aged 0 to 15 y residing in six regions covered by the EMBLEM study in six regions of northwestern Kenya, northeastern Tanzania, and northern Uganda (*SI Appendix*, Fig. S2) (20). The two regions in Uganda were transected by the Nile River but otherwise contiguous, as were the four regions in Kenya and Tanzania, which bordered Lake Victoria in both countries (*SI Appendix*, Fig. S2). These regions were targeted because they have high malaria incidence, which was confirmed through age-weighted prevalence of asymptomatic *P. falciparum* infection (40 to 55%) (39, 48–50) and because previous studies indicated a high eBL incidence in the population (20). The eBL cases analyzed in this study were ascertained in the EMBLEM study. Because case ascertainment started at different times of the calendar year in different areas (*SI Appendix*, Fig. S3), we calculated the amount of person-time at risk as the person time per fraction of each year during which case ascertainment was active in that region.

The population of children at risk of eBL at each census enumeration area (EA) was estimated using data obtained (by SMM for the EMBLEM study) from each country’s statistical bureau and interpolated at NCI, using district or regional average population growth rates as previously described (39, 48, 49). The EAs are referred to as a Parish in Uganda, a Ward in Tanzania, and Sub-location in Kenya, are comprised of an average of about 6 villages, and are nested hierarchically within the next-level administrative areas. Kenya and Uganda provided total and sex-specific population counts for each EA in 5-y age groupings (0 to 4, 5 to 9, 10 to 14, etc.) for the year of their national census (2009 in Kenya and 2002 and 2014 in Uganda). In addition, the bureaus also provided intercensal population projections for 10 y and the average population growth rates by district or region used to generate those projections. Single-year population counts for the whole country were also provided. The count of 15-y-olds was obtained by applying the national proportion of children aged 15 y in the 15 to 19-y age group to the count in the 15 to 19-y age group for each EA. The distribution of the national population in single-year age groups was applied to split the population at risk in a single year by age and sex. In Tanzania, the national statistical bureau provided population count data by age in single-year age groups as well as 5-y age groups by sex for each ward in the district. The Tanzanian bureau also provided population projections over a ten-year period or population growth rate for each district or region, which were used for additional interpolation in our study. These data were cross-checked at NCI and interpolated to estimate the number of children aged 0 to 15 y in the study area. For Uganda where data were obtained for 2002 and 2014 censuses, interpolation from 2002 census data through 2014 produced reasonable population estimates to the counts in the 2014 census (49).

The average population under observation per year was 8,541,872 (3,443,563 in Kenya; 5,820,176 in Tanzania; and 1,924,915 in Uganda), yielding a total population of 59,793,102 person-years that were contributed by children 0 to 15 y old (17,217,816 in Kenya; 29,100,881 in Tanzania; and 13,474,404 in Uganda) distributed spatially as shown in *SI Appendix*, Fig. S2 and temporally as shown in *SI Appendix*, Fig. S3*A*. Population data were aggregated to the district level (i.e., one administrative level below the region), which was used as the unit for spatial analysis to achieve better statistical stability.

#### *P. falciparum* Exposure.

We estimated the population exposure to *P. falciparum* using high-resolution age-standardized estimates of *Pf*PR2-10 from MAP (23), which is designated as a World Health Organization (WHO) Collaborating Centre in Geospatial Disease Modeling. Their data are the most reliable estimates of malaria burden (23, 50) and are used to inform malaria control and elimination efforts. The metric *Pf*PR2-10 is the estimated proportion of the population of children aged 2 to 10 y carrying blood-stage *P. falciparum* parasites and is considereda reliable index of transmission intensity (51).

[1] $$\begin{array}{c} {PfPR}_{2-10}\left(x,\;t\right)=\frac{PfEIR{\left(x,\;\;t-1\right)}^{0.119}}{(PfEIR{\left(x,\;\;t-1\right)}^{0.119}+\;\exp\left(0.523\right)}*12. \end{array}$$

Eq. **1** relates the PfPR2-10 of district *x* in year *t* to the PfEIR of district *x* in year *t−1* (1 y prior to *t*). The parameters are specific to children and the formula has been modified to estimate annual PfEIR instead of monthly.

[2] $$Y_{i,j}∼Negative\;Binomial\left(\lambda_{i,j},\;\phi \right),$$

$$E\left[Y_{i,j}\right]=\;\lambda_{i,j},$$

$$\mathrm{Var}\left[Y_{i,j}\right]=\frac{\lambda_{i,j}+\lambda_{i,j^{2}}}{\varphi },$$

$$\log\left(\lambda_{i,j}\right)\;=\;\beta_{0}\;+\;\;\beta X_{i,j}+\;\log\left(population_{i,j}\right)\;+\;\mu_{i}.$$

Eq. **2** represents the number of eBL cases, for the *i*th observation of the *j*th district as a function of *β*, a vector of estimated coefficients, and *X*, a vector of the covariates age, sex, country, year. The random effect, *μ_i_*, allows the estimated count of eBL cases to be systematically higher or lower in some districts.

We selected the *Pf*PR in children because it is correlated with the *Pf*EIR, which represents the number of infectious mosquito bites per person in a defined period (24). *Pf*PR2-10 is traditionally used to group malaria transmission into hypoendemic (<10%), mesoendemic (10 to 50%), and hyperendemic (50 to 75%) categories. Holoendemic malaria, representing the most intense transmission, is defined as *Pf*PR >75% in children aged 0 to 12 mo. This metric *Pf*PR2-10 is a relevant predictor for eBL because it covers the age range when exposure to *P. falciparum* is thought to modulate eBL risk and it is amenable to mathematical or statistical modeling (51). The MAP has estimated the annual values based on multiple country-specific surveillance datasets (23). We note that *Pf*PR2-10 does not cover the complete age range of the population we considered at-risk for eBL, i.e., 0 to 15 y, because it is designed to perform optimally as a reliable indicator of the force of infection in the community (24). It excludes children older than 10 y in whom acquired immunity is reasonably well established and acts a counterforce to reduce the probability of successful infection. We extracted estimated *Pf*PR2-10 for the period 2000 through 2017 at 5 km × 5 km resolution for the areas targeted by the EMBLEM study. For our analysis, *Pf*PR2-10 was aggregated at the district level by calculating a weighted *Pf*PR2-10 average based on the proportion of the district occupied by each 25 sq. km. data unit. We utilized the known relationship between *Pf*PR2-10 and *Pf*EIR (25) to calculate the annual *Pf*EIR using a previously defined logit-linear relationship modified to provide annual *Pf*EIR estimates (Eq. **1**, *SI Appendix*, Fig. S1 ). Then, assuming a transmission probability (i.e., mosquito-inoculated sporozoites lead to a blood-stage infection, which is considered the relevant exposure) of 10% (27, 28), we calculated a credible average annual number of incident infections per person, by age, year, and region. This estimate is used to examine the trends in estimated *P. falciparum* incidence by year and country (*SI Appendix*, Fig. S4) and its association with eBL.

To investigate whether endemicity of *P. falciparum* was correlated with eBL in time and space, we created a variable to capture the cumulative number of *P. falciparum* infections a child in a particular area acquires by summing the estimated infections for each annual birth cohort within each district. Because high-resolution *Pf*PR2-10 data have only been available from 2000 onward, this variable could not be estimated for children born before 1999 in Uganda or before 2001 in Kenya and Tanzania, so these birth cohorts are excluded from our analysis. Thus, eBL outcomes were considered only for those birth cohorts whose cumulative infections over their entire lifespan could be estimated. For a child who developed eBL, the cumulative number of estimated *P. falciparum* infections at the age of diagnosis was considered their exposure, whereas for children who did not develop eBL, their number of estimated infections continued to increase up to a maximum determined by their age. The 552 children diagnosed with eBL are not removed from the at-risk population because their person-time was considered negligible relative to the population denominators. We note that although the number of cumulative *P. falciparum* infections increases with age, this increase does not result in increased malaria-related morbidity or mortality because children generally develop premunition (anti-disease and anti-parasite immunity), which reduces the risk for malaria and ultimately causes the *Pf*PR2-10 to plateau and decrease (52–54).

#### eBL Case Data.

We used eBL case data prospectively ascertained by the EMBLEM study from the defined study areas from 2010 through 2016 (20) as the outcome data. Cases of eBL were confirmed by histology or cytology (61%) whenever possible (20) and, when not possible, by consistent clinical features, imaging, and laboratory results that support the presumptive diagnosis of a typical eBL case. The study enrollment started in Uganda in November 2010, was introduced in July 2012 in Kenya and Tanzania, and continued through September 2016 in all three countries (*SI Appendix*, Fig. S3). The EMBLEM study implemented regular public awareness campaigns to sensitize the community about eBL and encourage referral of suspected cases to collaborating hospitals with the capacity to diagnose and treat eBL in the six regions. The study facilitated the diagnosis and treatment of patients referred to the hospitals. Information on which cases were included in the analysis can be found in *SI Appendix*, Fig. S5. The EMBLEM study collected detailed information on the cases in this analysis, including geographic origin (20). Demographic characteristics of cases were compared across countries using ANOVA. Overall eBL incidence, expressed as cases per million per country, was calculated as the age-specific number of cases identified in that country divided by the total-age-specific person-time at risk in that country (see *Study Population*). The time trends in eBL incidence over the study period were examined to determine its correlation with *P. falciparum* incidence and the impact of awareness campaigns (*SI Appendix*, Fig. S4).

### Research Ethics.

Written informed consent was obtained from guardians of all study participants and assent from children >7 y old. Ethical approval for EMBLEM research was granted by Uganda Virus Research Institute (GC/127), Uganda National Council for Science and Technology (H816), Tanzania National Institute for Medical Research (NIMR/HQ/R.8c/Vol. IX/1023), Moi University/Moi Teaching and Referral Hospital (000536), and National Cancer Institute (10-C-N133) and did not completely overlap spatially.

Spatiotemporal Clustering of eBL and *P. falciparum* Incidence. District-level eBL incidence was calculated as the age-specific number of cases identified in that country divided by the total age-specific person-time at risk in that country (see *Study Population*). Overall eBL incidence in each district was calculated as the number of cases per million children in each age and sex category, per year, and per district and was expressed as cases per million children. Within each 5-y age group, we made the simplifying assumption that every year of age represented an equal share of the population. We investigated trends in eBL and cumulative estimated *P. falciparum* incidence by first producing maps of the spatiotemporal distribution of both conditions, averaged over the study period, and calculated the Spearman correlation coefficient between average eBL incidence and average cumulative *P. falciparum* infections across all 49 districts. Next, we identified patterns of spatial clustering of cumulative estimated *P. falciparum* incidence and of eBL incidence using Moran’s Local Indicator of Spatial Autocorrelation (LISA) statistics (55), which identified spatial clusters of high or low values within each calendar year. We aggregated the number of times each district had significantly higher or lower values than its surrounding districts. This analysis was repeated with aggregated eBL incidence and cumulative *P. falciparum* incidence over the entire study period. We then employed a bivariate Moran’s *I* statistical test to describe the relationship between the eBL incidence of a district and the cumulative estimated *P. falciparum* incidence in the same and surrounding districts (56) within each calendar year and aggregated over the study period. In the annual analysis, the number of concordant and discordant bivariate Moran’s I values per district was summarized over all the years of the study. All LISA statistical analyses were performed separately for each spatially contiguous region.

### Spatial Regression Analysis of eBL Risk.

We used our spatial estimates of age-specific lifetime *P. falciparum* infections to model the rate of eBL diagnosis by age and sex, in a given country and district, during a given year, conditional on no prior eBL diagnosis. Because eBL is rare, and the incidence rate is highly variable across locations, a negative binomial regression was used (Eq. **2**, *SI Appendix*, Fig. S1, see p. 37) to account for possible overdispersion. A random intercept was included for each district because there are repeated measurements of each spatial unit over multiple years. Fixed effects were also included for the relationship between age, sex, country, and calendar year. To ensure that our cumulative-exposure metric was preferable to a cross-sectional measurement of malaria exposure, we compared the performance of this model to those using cross-sectional annual exposures by age and location. Results from this analysis are described in the Appendix. All models were fitted using the “brms” package for Bayesian hierarchical regression modeling for R 4.0.0, using default priors for both regression and variance parameters (57, 58). Code for the analysis can be found at https://github.com/broenk/eBL. Malariometric data are available through the MAP, and data on eBL cases can be obtained on request from SMM.

## Supplementary Material

Appendix 01 (PDF)

## Data Availability

All Rode has been deposited in Github (https://github.com/broenk/eBL). Some study data available (data on eBL cases are considered protected health information and cannot be made publicly available. Investigators may request controlled access to the data. All code used for analysis and malaria data are publicly available and will be shared via a Github repository). All malaria data is available through the Malaria Atlas Project (https://malariaatlas.org/).

## References

[1] E. M. Molyneux , Burkitt’s lymphoma. The Lancet **379**, 1234–1244 (2012). 10.1016/S0140-6736(11)61177-X 22333947

[2] K. Basso, R. Dalla-Favera, Germinal centres and B cell lymphomagenesis. Nat. Rev. Immunol. **15**, 172–184 (2015). 25712152 10.1038/nri3814

[3] V. Bouvard , Carcinogenicity of malaria and of some polyomaviruses. Lancet Oncol. **13**, 339–340 (2012). 22577663 10.1016/s1470-2045(12)70125-0

[4] L. M. Carpenter , Antibodies against malaria and Epstein-Barr virus in childhood Burkitt lymphoma: A case-control study in Uganda. Int. J. Cancer **122**, 1319–1323 (2008). 18000823 10.1002/ijc.23254

[5] N. Mutalima , Associations between Burkitt lymphoma among children in Malawi and infection with HIV, EBV and malaria: Results from a case-control study. PloS One **3**, e2505 (2008). 18560562 10.1371/journal.pone.0002505 PMC2423475

[6] D. F. Robbiani , Plasmodium infection promotes genomic instability and AID-dependent B cell lymphoma. Cell **162**, 727–737 (2015). 26276629 10.1016/j.cell.2015.07.019 PMC4538708

[7] B. M. Grande , Genome-wide discovery of somatic coding and noncoding mutations in pediatric endemic and sporadic Burkitt lymphoma. Blood **133**, 1313–1324 (2019). 30617194 10.1182/blood-2018-09-871418 PMC6428665

[8] J. J. Rainey , Spatial clustering of endemic Burkitt’s lymphoma in high-risk regions of Kenya. Int. J. Cancer **120**, 121–127 (2007). 17019706 10.1002/ijc.22179

[9] J. J. Rainey , Spatial distribution of Burkitt’s lymphoma in Kenya and association with malaria risk. Trop. Med. Int. Health **12**, 936–943 (2007). 17697088 10.1111/j.1365-3156.2007.01875.x

[10] E. Williams, N. Day, A. Geser, Seasonal variation in onset of burkitt’s lymphoma in the West Nile district of Uganda. The Lancet **304**, 19–22 (1974). 10.1016/s0140-6736(74)91350-6 4134408

[11] D. P. Burkitt, Etiology of Burkitt’s lymphoma—An alternative hypothesis to a vectored virus. J. Natl. Cancer Inst. **42**, 19–28 (1969), 10.1093/jnci/42.1.19. 4303830

[12] J. A. Nájera, M. González-Silva, P. L. Alonso, Some lessons for the future from the Global malaria eradication programme (1955–1969). PLoS Med. **8**, e1000412 (2011). 21311585 10.1371/journal.pmed.1000412 PMC3026700

[13] M. K. Smatti , Epstein- Barr virus epidemiology, serology, and genetic variability of LMP-1 oncogene among healthy population: An update. Front. Oncol. **8**, 211 (2018). 29951372 10.3389/fonc.2018.00211 PMC6008310

[14] S. Bhatt , The effect of malaria control on Plasmodium falciparum in Africa between 2000 and 2015. Nature **526**, 207–211 (2015). 26375008 10.1038/nature15535 PMC4820050

[15] R. J. Biggar, F. K. Nkrumah, Burkitt’s lymphoma in Ghana: Urban-rural distribution, time—space clustering and seasonality. Int. J. Cancer **23**, 330–336 (1979). 437917 10.1002/ijc.2910230310

[16] R. H. Morrow, M. C. Pike, P. G. Smith, J. L. Ziegler, A. Kisuule, Burkitt’s lymphoma: A time-space cluster of cases in Bwanba County of Uganda. BMJ **2**, 491–492 (1971). 4325452 10.1136/bmj.2.5760.491 PMC1795979

[17] S. M. Mbulaiteye , African Burkitt’s lymphoma: Could collaboration with HIV-1 and malaria programmes reduce the high mortality rate? Lancet Lond. Engl. **375**, 1661–1663 (2010). 10.1016/S0140-6736(10)60134-1 20452522

[18] J. Siemiatycki, G. Brubaker, A. Geser, Space-time clustering of Burkitt’s lymphoma in East Africa: Analysis of recent data and a new look at old data. Int. J. Cancer **25**, 197–203 (1980). 7390649 10.1002/ijc.2910250206

[19] A. Geser, G. Brubaker, C. C. Draper, Effect of a malaria suppression program on the incidence of African Burkitt’s lymphoma. Am. J. Epidemiol. **129**, 740–752 (1989). 2923122 10.1093/oxfordjournals.aje.a115189

[20] S. Peprah , Risk factors for Burkitt lymphoma in East African children and minors: A case-control study in malaria-endemic regions in Uganda, Tanzania and Kenya. Int. J. Cancer **146**, 953–969 (2020). 31054214 10.1002/ijc.32390 PMC6829037

[21] R. W. Snow , The prevalence of Plasmodium falciparum in sub-Saharan Africa since 1900. Nature **550**, 515–518 (2017). 29019978 10.1038/nature24059 PMC5660624

[22] L. Hämmerl, M. Colombet, R. Rochford, D. M. Ogwang, D. M. Parkin, The burden of Burkitt lymphoma in Africa. Infect. Agent. Cancer **14**, 17 (2019). 31388351 10.1186/s13027-019-0236-7 PMC6670145

[23] D. J. Weiss , Mapping the global prevalence, incidence, and mortality of Plasmodium falciparum, 2000–17: A spatial and temporal modelling study. The Lancet **394**, 322–331 (2019). 10.1016/S0140-6736(19)31097-9 PMC6675740 31229234

[24] D. L. Smith, J. Dushoff, R. W. Snow, S. I. Hay, The entomological inoculation rate and Plasmodium falciparum infection in African children. Nature **438**, 492–495 (2005). 16306991 10.1038/nature04024 PMC3128496

[25] B. Amoah , Identifying Plasmodium falciparum transmission patterns through parasite prevalence and entomological inoculation rate. eLife **10**, e65682 (2021). 34672946 10.7554/eLife.65682 PMC8530514

[26] T. Reiker , Emulator-based Bayesian optimization for efficient multi-objective calibration of an individual-based model of malaria. Nat. Commun. **12**, 7212 (2021). 34893600 10.1038/s41467-021-27486-z PMC8664949

[27] T. S. Churcher , Probability of transmission of malaria from mosquito to human is regulated by mosquito parasite density in naïve and vaccinated hosts. PLoS Pathog. **13**, e1006108 (2017). 28081253 10.1371/journal.ppat.1006108 PMC5230737

[28] T. S. Churcher, J.-F. Trape, A. Cohuet, Human-to-mosquito transmission efficiency increases as malaria is controlled. Nat. Commun. **6**, 6054 (2015). 25597498 10.1038/ncomms7054 PMC4309425

[29] J. F. Namuganga , The impact of stopping and starting indoor residual spraying on malaria burden in Uganda. Nat. Commun. **12**, 2635 (2021). 33976132 10.1038/s41467-021-22896-5 PMC8113470

[30] C. M. Cote, Ongoing long-lasting insecticide-treated net distribution efforts are insufficient to maintain high rates of use among children in rural Uganda. medRxiv; 2021. 10.1101/2021.02.26.21252527.

[31] S. Ruybal-Pesántez, Age-specific patterns of DBLαvar diversity can explain why residents of high malaria transmission are as remain susceptible to Plasmodium falciparum blood stage infection throughout life. Int. J. Parasitol. **52**, 721–731 (2022), 10.1016/j.ijpara.2021.12.001. 35093396 PMC9339046

[32] V. Yman , Distinct kinetics of antibodies to 111 Plasmodium falciparum proteins identifies markers of recent malaria exposure. Nat. Commun. **13**, 331 (2022). 35039519 10.1038/s41467-021-27863-8 PMC8764098

[33] P. Aka , Endemic Burkitt lymphoma is associated with strength and diversity of Plasmodium falciparum malaria stage-specific antigen antibody response. Blood **122**, 629–635 (2013). 23645841 10.1182/blood-2012-12-475665 PMC3731925

[34] A. G. Buchwald , Association between age and Plasmodium falciparum infection dynamics. Am. J. Epidemiol. **188**, 169–176 (2019). 30252032 10.1093/aje/kwy213 PMC6321803

[35] J. E. Coalson , Simulation models predict that school-age children are responsible for most human-to-mosquito Plasmodium falciparum transmission in southern Malawi. Malar. J. **17**, 147 (2018). 29615044 10.1186/s12936-018-2295-4 PMC5883608

[36] S. Portugal , Host-mediated regulation of superinfection in malaria. Nat. Med. **17**, 732–737 (2011). 21572427 10.1038/nm.2368 PMC4200394

[37] A. Derkach , Associations between IgG reactivity to Plasmodium falciparum erythrocyte membrane protein 1 (PfEMP1) antigens and Burkitt lymphoma in Ghana and Uganda case-control studies. EBioMedicine **39**, 358–368 (2019). 30579868 10.1016/j.ebiom.2018.12.020 PMC6355394

[38] G. Brubaker, A. Geser, M. C. Pike, Burkitt’s lymphoma in the North Mara district of Tanzania 1964–70: Failure to find evidence of time-space clustering in a high risk isolated rural area. Br. J. Cancer **28**, 469–472 (1973). 4758376 10.1038/bjc.1973.175 PMC2008926

[39] S. Peprah , A cross-sectional population study of geographic, age-specific, and household risk factors for asymptomatic Plasmodium falciparum malaria infection in Western Kenya. Am. J. Trop. Med. Hyg. **100**, 54–65 (2019). 30457091 10.4269/ajtmh.18-0481 PMC6335891

[40] K. F. Schulz, D. A. Grimes, Case-control studies: Research in reverse. Lancet Lond. Engl. **359**, 431–434 (2002). 10.1016/S0140-6736(02)07605-5 11844534

[41] D. Burkitt, A sarcoma involving the jaws in African children. Br. J. Surg. **46**, 218–223 (1958). 13628987 10.1002/bjs.18004619704

[42] F. Okongo, D. M. Ogwang, B. Liu, D. Maxwell Parkin, Cancer incidence in Northern Uganda (2013–2016). Int. J. Cancer **144**, 2985–2991 (2019). 30536374 10.1002/ijc.32053

[43] L. S. Redmond , Endemic Burkitt lymphoma: A complication of asymptomatic malaria in sub-Saharan Africa based on published literature and primary data from Uganda, Tanzania, and Kenya. Malar. J. **19**, 239 (2020). 32718346 10.1186/s12936-020-03312-7 PMC7385955

[44] S. Gupta, R. W. Snow, C. A. Donnelly, K. Marsh, C. Newbold, Immunity to non-cerebral severe malaria is acquired after one or two infections. Nat. Med. **5**, 340–343 (1999). 10086393 10.1038/6560

[45] J. T. Griffin , Gradual acquisition of immunity to severe malaria with increasing exposure. Proc. Biol. Sci. **282**, 20142657 (2015). 25567652 10.1098/rspb.2014.2657 PMC4309004

[46] H. Hjalgrim, J. Friborg, M. Melbye, “The epidemiology of EBV and its association with malignant disease” in Human Herpesviruses: Biology, Therapy, and Immunoprophylaxis, A. Arvin , Eds. (Cambridge University Press, 2007). 21348109

[47] The “World malaria report 2019” at a glance World HealthOrganization https://www.who.int/news-room/feature-stories/detail/world-malaria-report-2019 (Accessed 15 June 2022).

[48] S. Peprah , A population-based study of the prevalence and risk factors of low-grade Plasmodium falciparum malaria infection in children aged 0–15 years old in northern Tanzania. Trop. Med. Int. Health TM IH **24**, 571–585 (2019). 30843638 10.1111/tmi.13225 PMC6499672

[49] M. Maziarz , Age and geographic patterns of Plasmodium falciparum malaria infection in a representative sample of children living in Burkitt lymphoma-endemic areas of northern Uganda. Malar. J. **16**, 124 (2017). 28320389 10.1186/s12936-017-1778-z PMC5360076

[50] U. N. Nakakana, I. A. Mohammed, B. O. Onankpa, R. M. Jega, N. M. Jiya, A validation of the Malaria Atlas Project maps and development of a new map of malaria transmission in Sokoto, Nigeria: a cross-sectional study using geographic information systems. Malar. J. **19**, 149 (2020). 32268904 10.1186/s12936-020-03214-8 PMC7140379

[51] D. L. Smith, C. A. Guerra, R. W. Snow, S. I. Hay, Standardizing estimates of the Plasmodium falciparum parasite rate. Malar. J. **6**, 131 (2007). 17894879 10.1186/1475-2875-6-131 PMC2072953

[52] J. W. G. Addy , 10-year longitudinal study of malaria in children: Insights into acquisition and maintenance of naturally acquired immunity. Wellcome Open Res. **6**, 79 (2022). 35141425 10.12688/wellcomeopenres.16562.1 PMC8822141

[53] T. Smith, I. Felger, M. Tanner, H. P. Beck, Premunition in Plasmodium falciparum infection: Insights from the epidemiology of multiple infections. Trans. R. Soc. Trop. Med. Hyg. **93** (suppl. 1), 59–64 (1999). 10450428 10.1016/s0035-9203(99)90329-2

[54] C. Rogier, D. Commenges, J. F. Trape, Evidence for an age-dependent pyrogenic threshold of Plasmodium falciparum parasitemia in highly endemic populations. Am. J. Trop. Med. Hyg. **54**, 613–619 (1996). 8686780 10.4269/ajtmh.1996.54.613

[55] L. Anselin, Local Indicators of Spatial Association—LISA. Geogr. Anal. **27**, 93–115 (1995).

[56] S.-I. Lee, Developing a bivariate spatial association measure: An integration of Pearson’s r and Moran’s I. J. Geogr. Syst. **3**, 369–385 (2001).

[57] R Development Core Team, R: A Language and Environment for Statistical Computing Journal of Statistical Software (2012). **100(5)**, 1–54. 10.18637/jss.v100.i05.

[58] P.-C. Bürkner, brms: An R Package for Bayesian Multilevel Models Using Stan. J. Stat. Softw. **80**, 1–28 (2017).

