# Supplementary material for: Burkitt lymphoma risk shows geographic and temporal associations with Plasmodium falciparum infections in Uganda, Tanzania, and Kenya
Source: Proc Natl Acad Sci U S A. 2023 Jan 3;120(2):e2211055120. doi: 10.1073/pnas.2211055120 (PMC9926229; doi:10.1073/pnas.2211055120)
Supplement: Supplementary file 1 — Appendix 01 (PDF) [file pnas.2211055120.sapp.pdf]

PNAS
www.pnas.org

Supporting Information for
Burkitt Lymphoma risk shows geographic and temporal associations with Plasmodium falciparum infections in Uganda, Tanzania, and Kenya

Kelly Broen[1,2], Joey Dickens[1,2], Rob Trangucci[3], Martin D. Ogwang[4], Constance N. Tenge[5], Nestory Masalu[6], Steven J. Reynolds[7], Esther Kawira[8], Patrick Kerchan[9], Pamela A. Were[5], Robert T. Kuremu[5], Walter N Wekesa[5], Tobias Kinyera[4], Isaac Otim[4], Ismail D. Legason[9], Ian D. Buller[10,11], Leona W. Ayers[12], Kishor Bhatia[10], Robert J. Biggar[10], James J. Goedert[10], Mark L. Wilson[1], Sam M. Mbulaiteye[10], and Jon Zelner[1,2]

1. Department of Epidemiology, University of Michigan School of Public Health, Ann Arbor, MI, USA
2. Center for Social Epidemiology and Population Health, University of Michigan School of Public Health, Ann Arbor, MI, USA
3. Dept. of Statistics, University of Michigan, Ann Arbor, MI, 48109, USA
4. EMBLEM Study, St. Mary's Hospital, Lacor, Gulu & African Field Epidemiology Network, Kampala, Uganda
5. EMBLEM Study, Moi University College of Health Sciences, Eldoret, Kenya & Academic Model Providing Access To Healthcare (AMPATH), Eldoret, Kenya
6. EMBLEM Study, Bugando Medical Center, Mwanza, Tanzania
7. Division of Intramural Research, National Institute of Allergy and Infectious Diseases, National Institutes of Health, Bethesda, Maryland, USA
8. EMBLEM Study, Shirati Health and Educational Foundation, Shirati, Tanzania
9. EMBLEM Study, Kuluva Hospital, Arua & African Field Epidemiology Network, Kampala, Uganda
10. Division of Cancer Epidemiology and Genetics, National Cancer Institute, National Institutes of Health, Bethesda, Maryland, USA
11. Cancer Prevention Fellowship Program, Division of Cancer Prevention, National Cancer Institute, National Institutes of Health, Bethesda, Maryland, USA
12. Department of Pathology, The Ohio State University, Columbus, Ohio, USA

Corresponding Authors: Kelly Broen (Email: broenk@umich.edu) and Sam M. Mbulaiteye (Email: mbulaits@mail.nih.gov)

This PDF file includes:

Supplementary text
Figures S1 to S9
Table S1

Methods

Evaluating temporal trends in estimated P. falciparum incidence and eBL incidence

To evaluate temporal trends in estimated *P. falciparum* incidence, we used a gaussian regression model with a fixed effect for year and country and a random effect for district. Year was included as a factor. Although estimated P. falciparum incidence was much lower in 2016 than in 2000, it has not been decreasing monotonically and increased between 2015-2016. While Tanzania has seen a constant slow decline in estimated *P. falciparum* incidence, Uganda saw an initial decrease followed by a spike in 2009. In 2016, both Uganda and Kenya saw an increase in estimated P. falciparum incidence from 2015, although estimated *P. falciparum* incidence is still lower than in 2000.

To determine if eBL incidence decreased in the study regions over the study period, regardless of *P. falciparum*, we employed a negative binomial regression with fixed effect for year, country, age, and sex. A random effect was included for the district, and the population was included in an offset term. We found age, included as a factor, to have a statistically significant relationship with eBL. Using age 10 years as the reference group, children 3 years and under or over 14 years had a lower risk of eBL. Children age 11 years had the highest risk and children 4-14 years had no statistically significant risk difference from children age 10. Risk did not differ significantly by year over the study period, although Tanzania had a much lower risk ratio (RR: 0.25, 95% CI: 0.11 - 0.54) than Kenya, while Uganda and Kenya did not significantly differ. Males had a 71% higher risk ratio than females (95% CI: 1.45 - 2.03).

Modeling the Association between eBL and Annual P. falciparum

Additional models examined the effect of individual years of estimated *P. falciparum* incidence on eBL incidence. First, 11 negative binomial regressions were performed with the same covariates as the main model from the text. Instead of including the cumulative estimated number of infections, the average number of infections *n* years ago was included in 11 separate models (Figure A9). Next, the estimated number of *P. falciparum* infections from the past 10 years and the current year were included in the same model (Figure A10). The 11 individual models showed a statistically significant protective effect of *P. falciparum* incidence 1 and 2 years prior, while *P. falciparum* incidence 9- and 10-years prior was associated with increased eBL risk. However, a district's estimated *P. falciparum* incidence is strongly correlated from year to year, making it difficult to interpret these results. The model with all years included showed no statistically significant effects but is also likely skewed by the correlation of covariates. These models demonstrate that, on their own, individual annual estimates of *P. falciparum* burden are poor predictors of eBL incidence and indicate the need for a metric of malaria burden over the life course.

Assessing the sensitivity of the relationship between eBL and Annual P. falciparum

To determine the sensitivity of the relationship between eBL and the annual *P. falciparum* incidence given the uncertainty in the $PfPR_{2-10}$ estimates, we recreated the main analysis using both the lower and upper bounds of the 95% confidence intervals of the $PfPR_{2-10}$ estimate. Using the lower estimate of annual *P. falciparum* incidence, we found that for every 100 cumulative *P. falciparum* infections, the risk of eBL increases 215% (95% CI: 1.80 – 5.39). Using the higher estimates, 100 cumulative *P. falciparum*

infections is associated with a 3% increase in the risk of eBL (95% CI: 0.99 – 1.06). Although the highest estimates of $PfPR_{2-10}$ do not result in a statistically significant association between eBL and cumulative *P. falciparum* incidence, point estimates demonstrate a positive association and indicate that only extreme deviation from the mean estimates of $PfPR_{2\text{-}10}$ nullify the association.

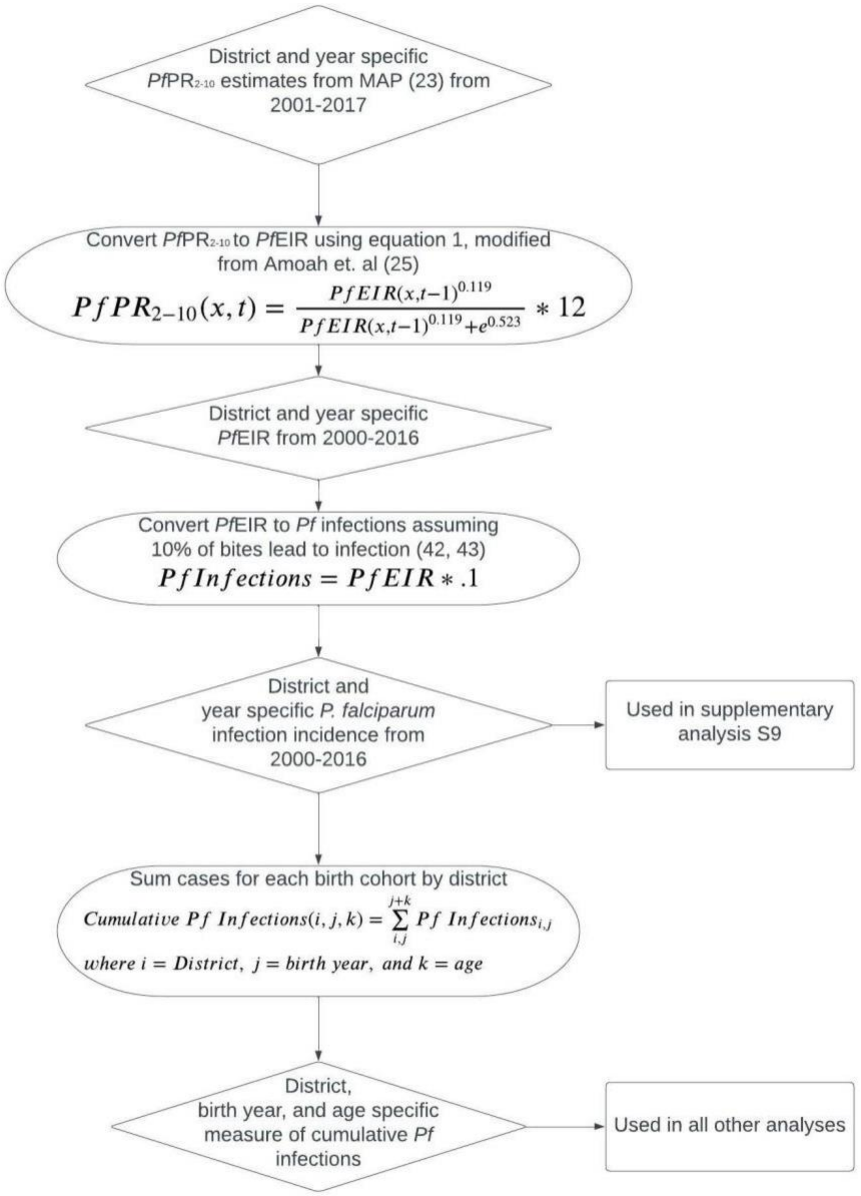

Fig. S1. Flow chart detailing the calculation of cumulative *P. falciparum* infections. In this flow chart, diamond shapes describe data and oval shapes describe the equations used to modify the data. Rectangles represent data used in analyses. Working down the flowchart, we start with annual district-level *Pf*PR_{2-10} converted to *Pf*EIR using a modified form of established relational equations from Amoah *et. al* (25). District- and year-specific *Pf*EIR is converted to the number of *P. falciparum* infections a child would experience in one year by assuming 10% of all bites lead to a *P. falciparum* infection. District and year specific estimated *P. falciparum* infections are used in the supplementary analysis described in Supp. Fig. S9. The number of annual infections is summed over the entire lifespan of each birth cohort, resulting in the cumulative number of *P. falciparum* infections a child has experienced in their lifetime which was used for all analyses in the main text.

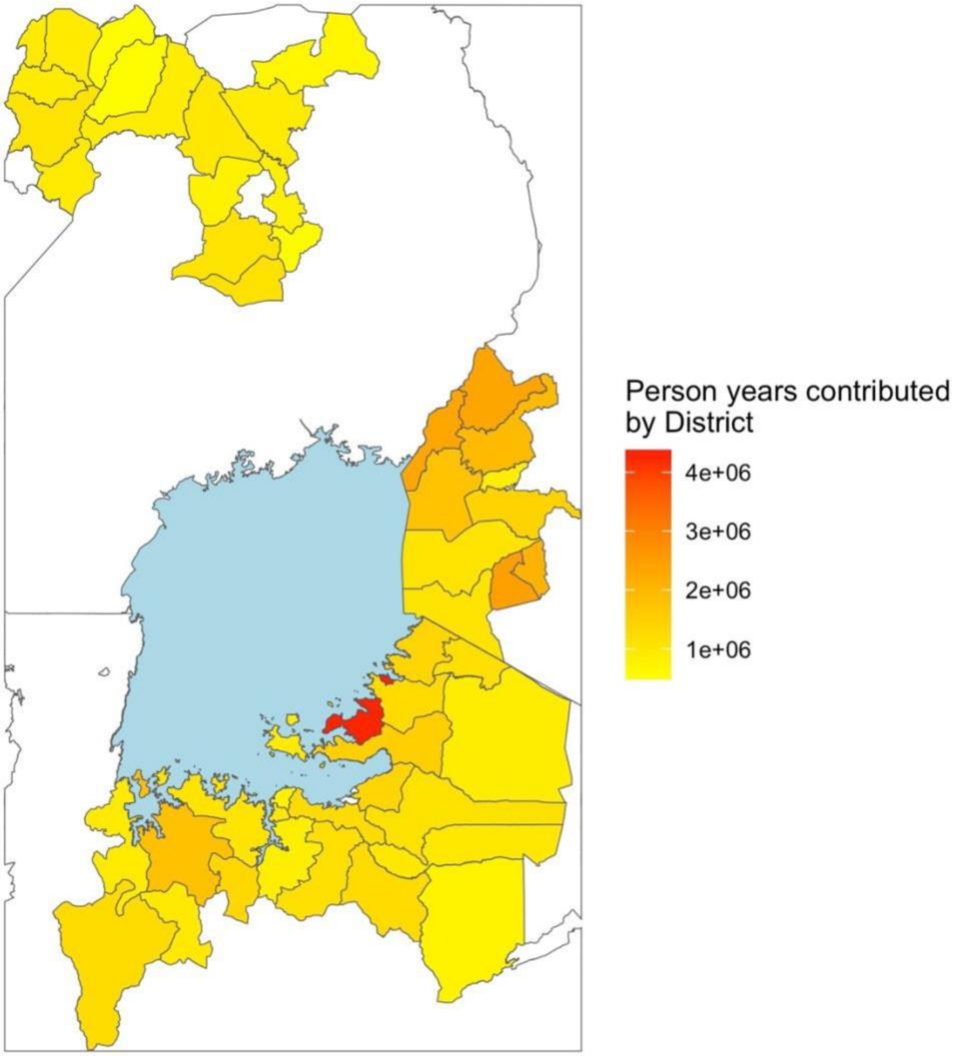

Fig. S2. Person-year density by District in the study. The amount of person-time in years contributed by each of the 49 districts over the study period is color coded to show population gradients. Uganda was included in the study from 2010 through September 2016, while Kenya and Tanzania only have data from 2012 through September 2016

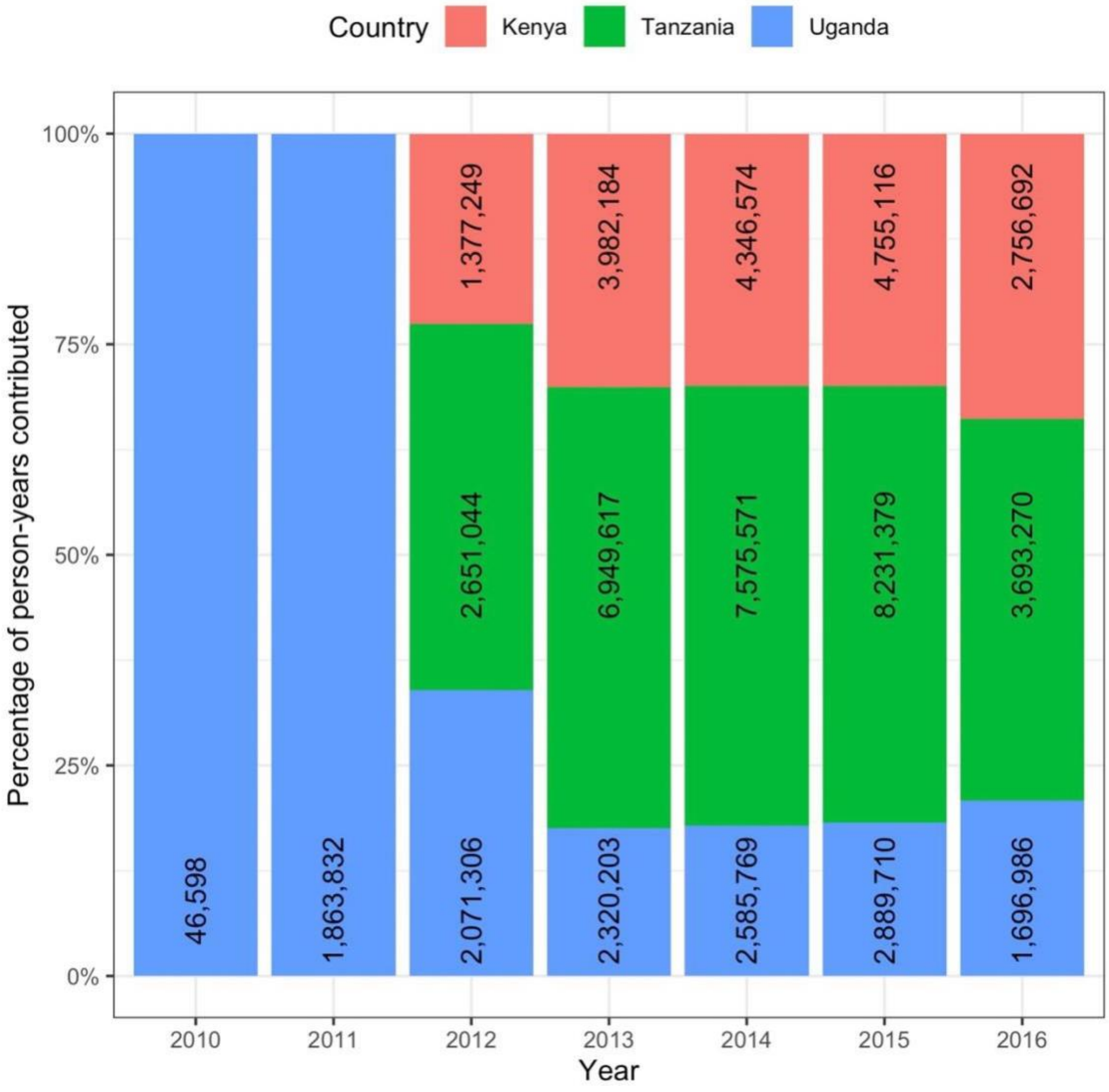

Fig. S3. Person-time contributed per year by country. Proportion of person-time contributed by all children aged 0-15 living in a given country during a given year of the study. Because case ascertainment began earlier in Uganda than in Kenya and Tanzania, all person-time in 2010 and 2011 came from case and non-case children in the study regions in Uganda. The number of person-years contributed by each country per year is shown in that country's proportion of person-years.

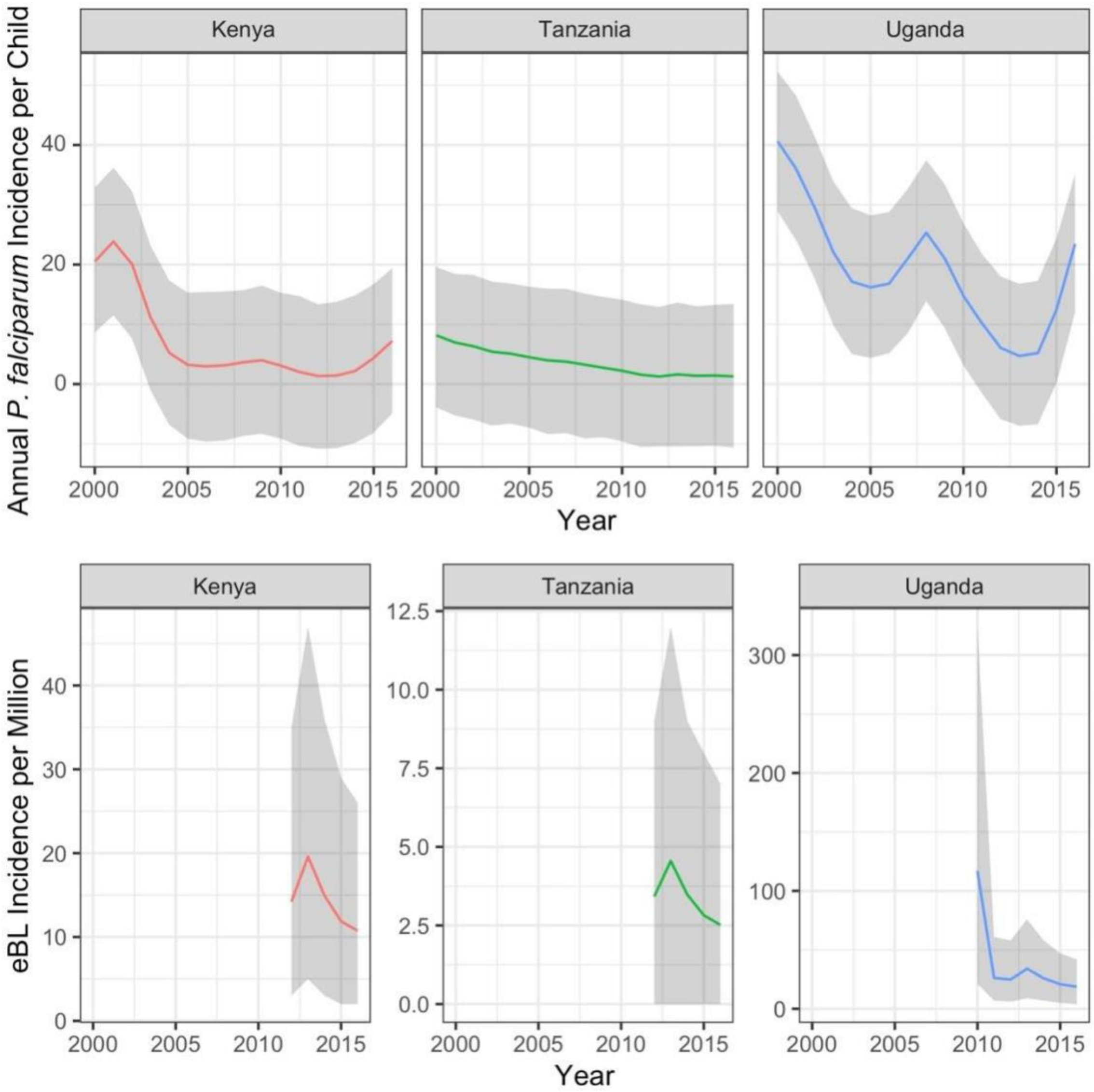

Fig. S4. Model estimated *P. falciparum* infection incidence and eBL incidence per million. The top panels show the modeled average number of estimated *P. falciparum* infections per year per child by country. The lower panels show modeled estimated eBL incidence throughout the study period by country. Note the different scales on the y-axis of the bottom panels. The 95% CIs are shown in grey.

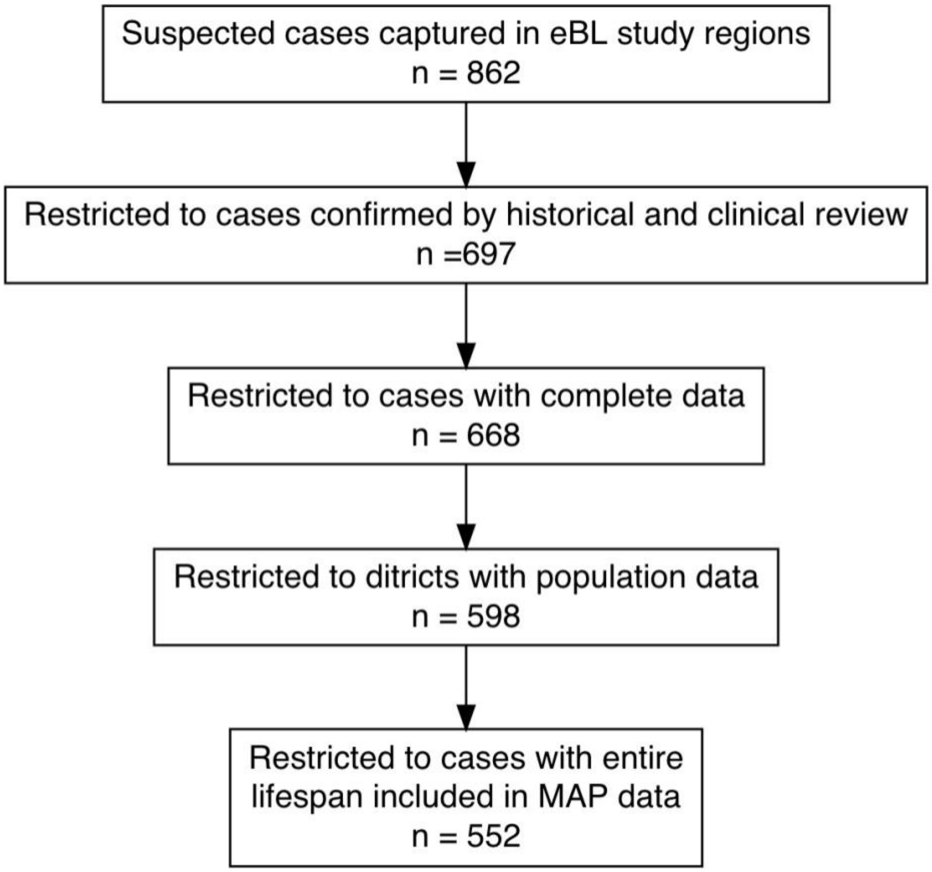

Fig. S5. Flow chart of eBL cases included in the analysis. Of the 862 suspected cases collected in EMBLEM, 552 were included in analysis due to exclusionary criteria.

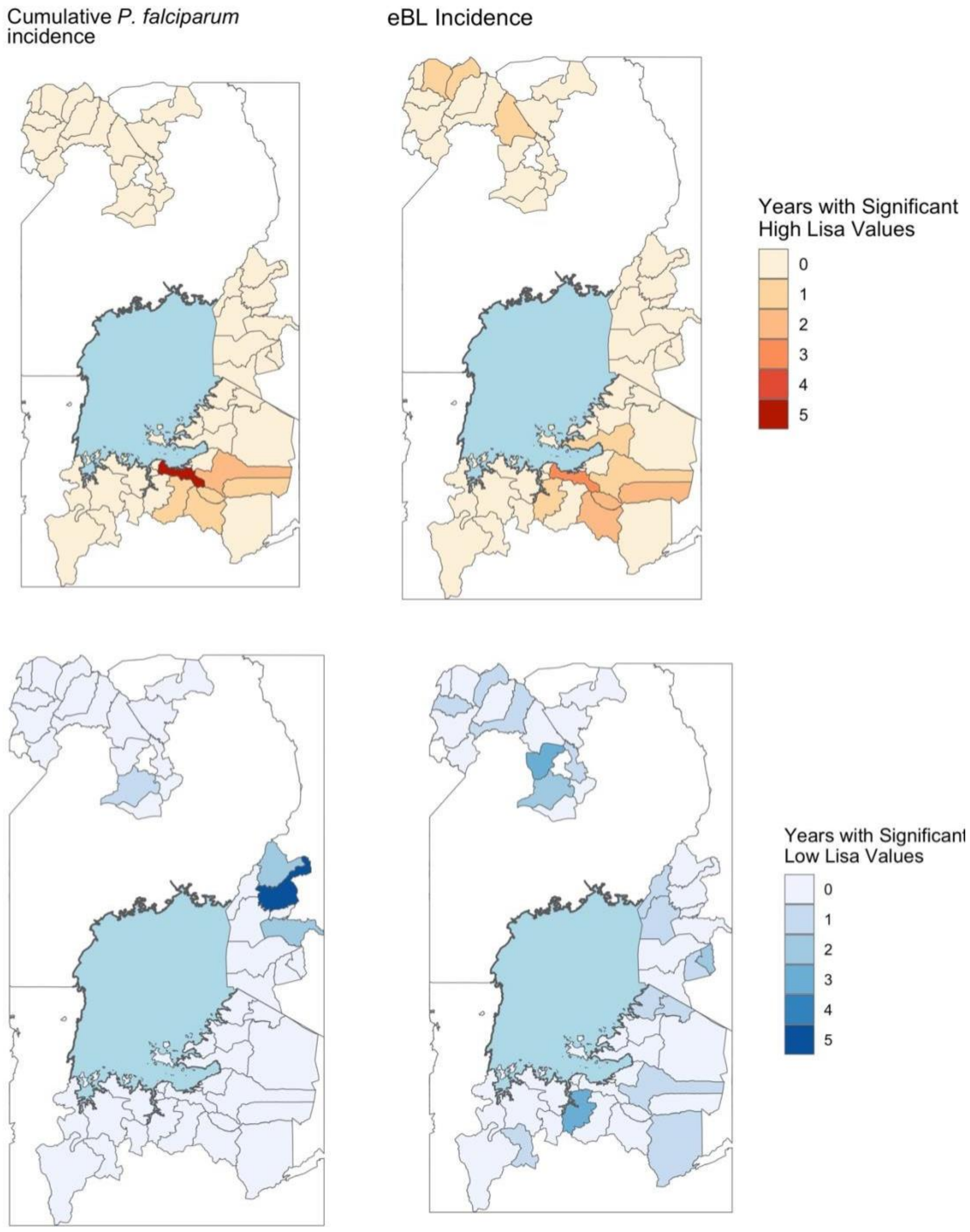

Fig. S6: Univariate Moran's LISA statistics for estimated cumulative *P. falciparum* incidence and eBL incidence during the study period (2010-2016). Univariate Moran's LISA statistics indicate clusters of high values of eBL and estimated *P. falciparum* incidence. The LISA values were calculated for each district given its annual eBL incidence throughout its time in the study and estimated cumulative *P. falciparum*

infections per child per year. The number of times a district had a significantly high or low value was summed and is displayed here.

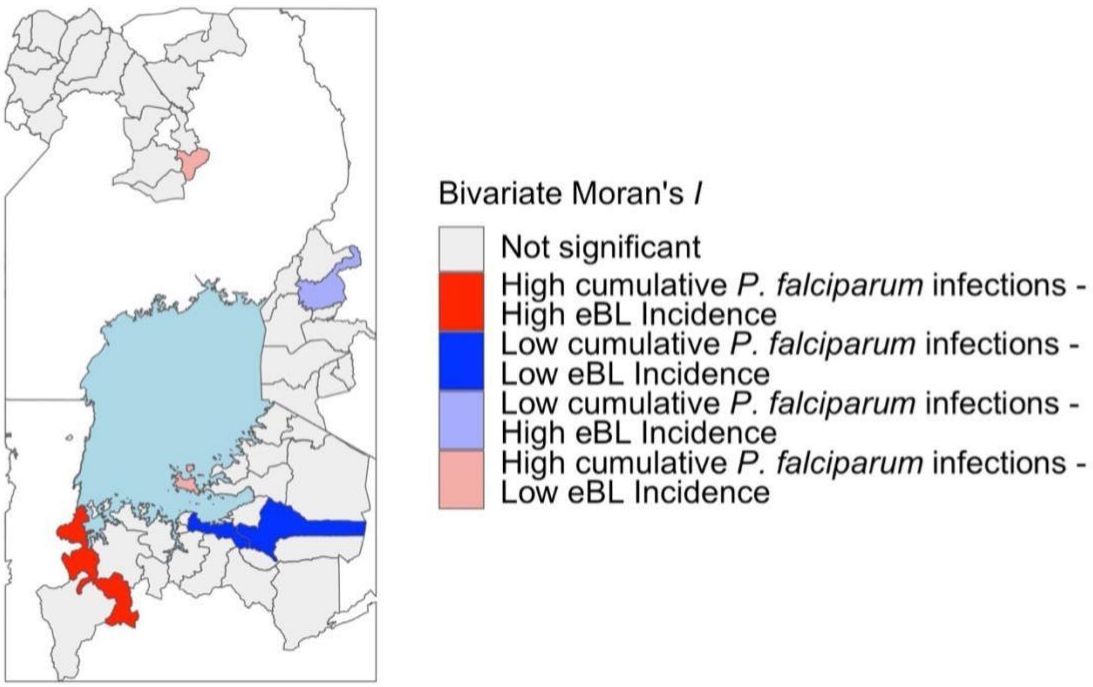

Fig. S7. Bivariate Moran's I LISA comparing average eBL incidence and average cumulative *P. falciparum* infections over the entire study period. The bivariate Moran's I LISA was calculated for each district comparing the average eBL incidence and average cumulative *P. falciparum* infections over the entire study period, demonstrating where similar values or dissimilar values cluster together.

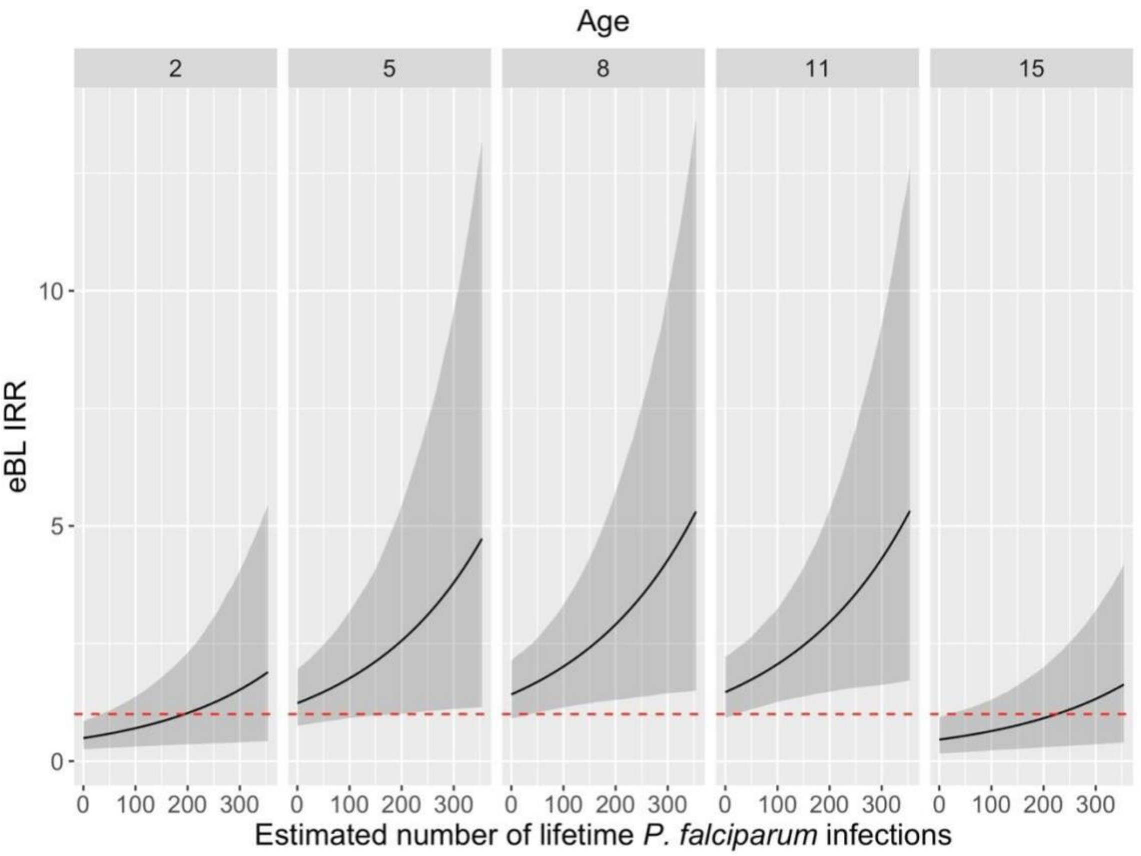

Fig. S8. eBL Incidence Rate Ratio with Increasing Cumulative *P. falciparum* Infections by Age. The IRR of eBL increases 39% for every 100 cumulative *P. falciparum* infections at all ages. Ages 2, 5, 8, 11, and 15 were selected to demonstrate the effect of age on risk. When compared to a 10 yo with 100 cumulative infections, the simulation shows that the IRR for eBL attains statistical significance (i.e., estimated IRR 95% lower confidence interval is above 1) among 5-11 yos, whereas by age 15, there is no statistically significant difference from the reference group regardless of *P. falciparum* exposure.

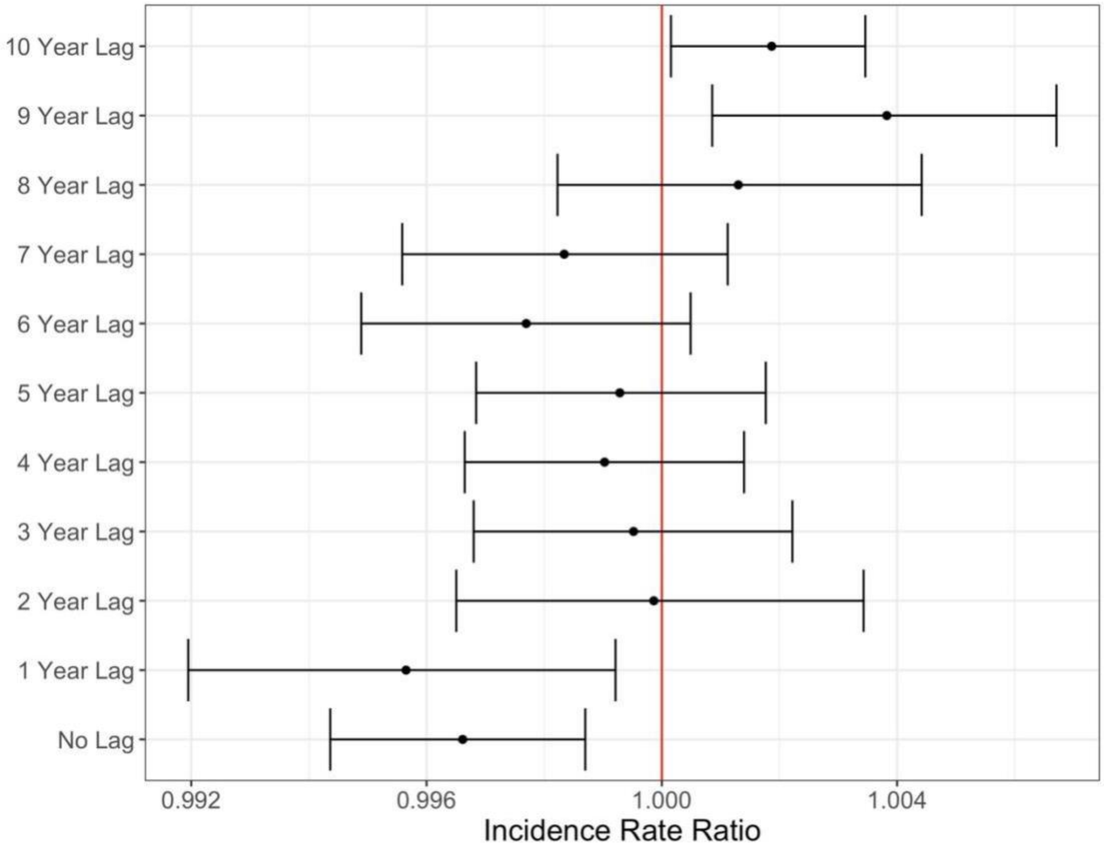

Fig. S9. Incidence Rate Ratios of lagged *P. falciparum* infection incidence at 0 through 10-year lags, with each lag included individually. The IRR associated with prior years' estimated *P. falciparum* incidence from 10 years ago until the present year when each variable is individually included its own model demonstrates a statistically significant protective effect of *P. falciparum* incidence from current year and 1 year prior. These findings demonstrate that a single year's measure of *P. falciparum* infection is inadequate to represent the lifetime *P. falciparum* burden, because children under two years old, who only have *P. falciparum* exposure in the current and previous year, are unlikely to develop eBL regardless of exposure burden, introducing a cohort effect that may confound results. Additionally, the immune response to *P. falciparum* alters the probability of successful infection in later years, leading to potential misclassification of the exposure and biased results. The *P. falciparum* incidences 9- and 10-years prior are associated with an increased IRR; however, these results should be interpreted with caution because many of the age groups with the highest eBL incidence (5-11 yo's) lack a 9- and 10-year lagged exposure.

Table S1: Results from negative binomial regression of eBL incidence and cumulative *P. falciparum* incidence, exponentiated to be interpreted as the incidence rate ratio

	Covariate	Estimate	95% Credible Interval
	Intercept	0.00	(0.00, 0.00)
	100 Malaria infections	1.39	(1.06, 1.81)
**Age**	0	0.00	(0.00, 0.01)
	1	0.09	(0.02, 0.26)
	2	0.48	(0.25, 0.86)
	3	0.74	(0.43, 1.30)
	4	1.36	(0.83, 2.18)
	5	1.22	(0.75, 1.96)
	6	1.40	(0.89, 2.24)
	7	1.26	(0.71, 1.95)
	8	1.42	(0.92, 2.20)
	9	1.44	(0.95, 2.27)
	10	**Ref**	
	11	1.48	(0.96, 2.29)
	12	0.92	(0.56, 1.46)
	13	0.63	(0.36, 1.07)
	14	0.65	(0.34, 1.20)
	15	0.43	(0.17, 0.99)
**Sex**	Female	**Ref**	
	Male	1.71	(1.43, 2.05)
**Country**	Kenya	**Ref**	

	Tanzania	0.25	(0.12, 0.50)
	Uganda	1.33	(0.61, 2.90)
**Year**	2010	**Ref**	
	2011	0.25	(0.09, 0.80)
	2012	0.26	(0.10, 0.82)
	2013	0.37	(0.14, 1.15)
	2014	0.30	(0.11, 0.91)
	2015	0.24	(0.09, 0.78)
	2016	0.21	(0.08, 0.66)
